# Clerodane-type Diterpene Glycosides from *Dicranopteris pedata*

**DOI:** 10.1007/s13659-021-00315-y

**Published:** 2021-06-05

**Authors:** Bei-Bei Gao, Yu-Fei Ou, Qin-Feng Zhu, Zhi-Ping Zhou, Zhen-Tao Deng, Meng Li, Qin-Shi Zhao

**Affiliations:** 1 Key Laboratory of Medicinal Chemistry for Natural Resource, Ministry of Education, School of Chemical Science and Technology, Yunnan University, Yunnan, 650091 Kunming, People’s Republic of China; 2 State Key Laboratory of Phytochemistry and Plant Resources in West China and Yunnan Key Laboratory of Natural Medicinal Chemistry, Kunming Institute of Botany, Chinese Academy of Sciences, Kunming, 650201 China; 3 College of Pharmacy, Guizhou Medical University, Guian New Area, Guizhou, 550025 China

**Keywords:** *Dicranopteris pedata*, Clerodane-type diterpene glycosides, ECD

## Abstract

Three new clerodane-type diterpene glycosides, (5*R*,6*S*,8*R*,9*S*,10*R*)-6-*O*-[*β*-d-glucopyranosyl-(1 → 4)-*α*-l-rhamnopyranosyl]cleroda-3,13(16),14-diene (**1**), (5*R*,6*S*,8*R*,9*S*,10*R*,13*S*)-6-*O*-[*β-*d-glucopyranosyl-(1 → 4)-*α*-l-rhamnopyranosyl]-2-ox-oneocleroda-3,13-dien-15-ol (**2**), (5*R*,6*S*,8*R*,9*S*,10*R*)-6-*O*-[*β*-d-glucopyranosyl-(1 → 4)-*α*-l-rhamnopyranosyl]-(13*E*)-2-oxoneocleroda-3,14-dien-13-ol (**3**), together with two known compounds **4** and **5** were isolated from *Dicranopteris pedata*. The structures of these compounds were elucidated by detailed spectroscopic analysis, and the absolute configuration of compound **2** was determined by ECD calculations. In addition, compound **1** exhibited weak inhibitory activities against SMMC-7721, MCF-7 and SW480.

**Supplementary Information:**

The online version contains supplementary material available at 10.1007/s13659-021-00315-y.

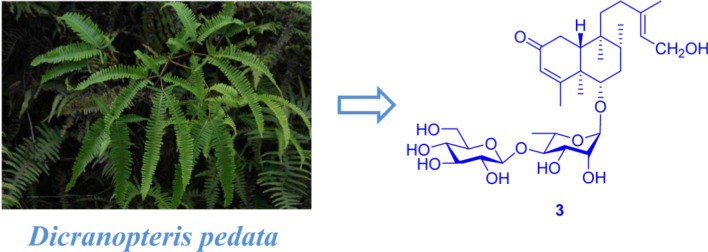

## Introduction

**Fig. 1:**
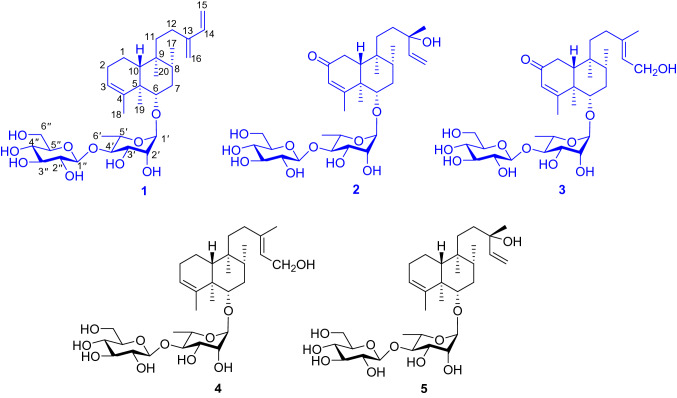
Chemical structures of compounds **1**–**5**

The genus *Dicranopteris* is consists of about 10 species, distributed mainly in Asia and widely in Japan, India, Vietnam and China. There are six species in China and they mainly distribute in the south of the Yangtze River such as Yunnan, Sichuan and Guizhou provinces [1]. The whole plant of *D. pedata* is commonly used as a folk medicine in ancient China for the treatment of hemorrhage, dysentery and empyrosis [2, 3]. Previous researches revealed a diversity of pharmacological properties of the extract of *D. pedata*, such as antioxidant, antibacterial, antinociceptive, anti-inflammatory and antipyretic activities [4–8]. Up to date, various secondary metabolites including flavonoids [9, 10], phenols [11], proanthocyanidins [12] and clerodane-type diterpene glycosides [10, 13] have been reported from *D. pedata*. Our previous investigation on this species revealed the presence of two oxygenated phenolic derivatives [14], dichotomains A and B and some clerodane-type diterpene glycosides, in which dichotomain A has exhibited weak anti-HIV activity [15, 16]. As part of our ongoing search for new bioactive metabolites from fern plants, a chemical investigation on the whole plant of *D. pedata* led to the discovery of three previously undescribed compounds (**1**–**3**) (Fig. 1) and two known analogues (compounds **4** and **5**) from the acetone extract [15, 16]. This paper describes the details of isolation, structure identification, and cytotoxicity of compounds **1**–**3**.

## Results and Discussion

**Fig. 2:**
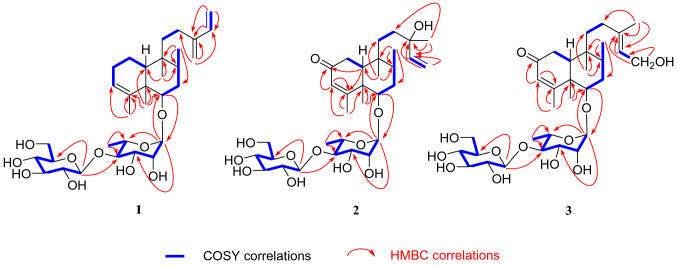
Key ^1^H–^1^H COSY and HMBC correlations of compounds **1**–**3**

Compound **1** was isolated as pale yellow powder. Its molecular formula was deduced as C_32_H_52_O_10_ by HRESIMS analysis (found *m*/*z* 619.3454 [M + Na]^+^, calcd for 619.3458), suggesting seven degrees of unsaturation. The ^13^C and ^1^H NMR spectra displayed two set of signals ascribed to a diterpene and two hexoses. Five methines (*δ*_C_ 103.2, 72.6, 72.7, 83.5, 68.8) and a methyl (*δ*_C_ 17.9) revealed the presence of rhamnose. In addition, the spectra displayed signals for a glucopyranosyl unit (five methines at *δ*_C_ 105.7, 76.1, 78.1, 71.5, 78.2 and a methylene at *δ*_C_ 62.7). Assignment of each glycosidic proton system was achieved by analysis of ^1^H-^1^H COSY, HMBC and HSQC spectra (Fig. 2). The ^1^H NMR spectrum of the diterpene displayed the presence of four methyls at *δ*_H_ 0.73 (s), 1.08 (s), 0.84 (d, *J* = 6.7 Hz) and 1.73 (d, *J* = 1.1 Hz, an olefinic methyl), and six olefinic protons at *δ*_H_ 5.24 (br s), 6.37 (dd, *J* = 17.6, 10.9 Hz), 5.20 (d, *J* = 17.6 Hz), 5.04 (d, *J* = 10.9 Hz), and 4.97 (2H, d, *J* = 4.4 Hz). The signal at *δ*_H_ 3.43 (dd, *J* = 11.2, 4.7 Hz) could be attributed to a proton on the oxygenated carbon. Twenty carbons of the diterpene could be classified to four methyls, seven methylenes (two olefinic at *δ*_C_ 113.3, 116.2), five methines (one oxygenated at *δ*_C_ 87.7; two olefinic at *δ*_C_ 123.6, 140.1) and four quaternary carbons (two olefinic at *δ*_C_ 144.6, 148.9). The structure of **1** was identified as a clerodane-type diterpenoid glycoside with two sugar moieties. Aforementioned 1D NMR resonances of **1** showed good agreement with the known compound **5** [16], except for the presence of one olefinic methylene and one olefinic quaternary carbon, together with the absence of a methyl and hydroxyl. The difference of compounds **1** and **5** lie in C-13, C-14, C-15 and C-16. The HMBC correlations (Fig. 2) from H_2_-16 (*δ*_H_ 4.97) to C-12 (*δ*_C_ 25.5), C-13 (*δ*_C_ 148.9), C-14 (*δ*_C_ 140.1), and H_2_-15 (*δ*_H_ 5.20, 5.04) to C-13 and C-14, accompanied with the ^1^H-^1^H COSY correlations (Fig. 2) between H-14 (*δ*_H_ 6.37) and H_2_-15 (*δ*_H_ 5.20, 5.04) confirmed the existence of conjugated diene at C-13/C-16 and C-14/C-15. Thus the planar structure of compound **1** was determined.

The relative configurations of *α-*rhamnopyranosyl and *β-*glucopyranosyl moiety were identified by the coupling constants of their anomeric protons of the rhamnopyranosyl (H-1ʹ, *δ*_H_ 4.79, *J* = 1.5 Hz) and glucopyranosyl (H-1*ʺ δ*_H_ 4.59, *J* = 7.8 Hz), respectively. The location of the sugar chain was determined at C-6 of the aglycone by HMBC correlations (Fig. 2) from the anomeric proton (H-1ʹ, *δ*_H_ 4.79) of the rhamnopyranosyl unit to C-6 (*δ*_C_ 87.7). HMBC correlation from H-1ʺ (*δ*_H_ 4.59, *J* = 7.8 Hz) to C-4ʹ (*δ*_C_ 83.5) defined a glucopyranosyl (1 → 4) rhamnosyl linkage. The absolute configurations of sugar moieties were determined by the experiments of acid hydrolysis, together with sugar derivatization and analysis with HPLC [16]. The retention times of the product in the test sample are consistent with that of standard sugar derivatives in HPLC (Rha derivative: *t*_R_ = 6.68 min; Glc derivative: *t*_R_ = 12.78 min), which gave d-glucose and l-rhamnose.

The relative configuration of **1** was assigned by analysis of its ROESY data (Fig. 4). The correlations of H-6/H-8, and H-6/H-10 indicated that they are spatially close and were thus assigned arbitrarily as *β*-oriented. Consequently, the correlations of CH_3_-19/CH_3_-20 revealed that they are cofacial and adopt a *α*-orientation. The correlation of H-16/H-14 disclosed the configuration of s-(*E*)-13(16),14-diene. The similarity of the NMR results between **1** and **5** suggests the same (5*R*,6*S*,8*R*,9*S*,10*R*) absolute configurations for the aglycone as previously reported. Finally, the structure of **1** was determined as (5*R*,6*S*,8*R*,9*S*,10*R*)-6-*O*-[*β*-D-glucopyranosyl-(1 → 4)-*α*-l-rhamnopyranosyl]cleroda-3,13(16),14-diene.

Compound **2** was purified as pale yellow powder with a molecular formula of C_32_H_52_O_12_, which was revealed by the [M + Na]^+^ ion at *m/z* 651.3355 (calcd for 651.3357), implying seven indices of hydrogen deficiency. The analysis of 1D NMR spectrum revealed the presence of a diterpene and two hexoses. Each glycosidic proton system was confirmed by analysis of ^1^H-^1^H COSY, HMBC and HSQC (Fig. 2). The ^1^H NMR spectrum of the diterpene displayed the presence of five methyls, one olefinic methylene at *δ*_H_ 5.16 (dd, *J* = 17.4, 1.5 Hz) and 5.03 (dd, *J* = 10.8, 1.5 Hz), two olefinic at *δ*_H_ 5.69 (s), 5.84 (dd, *J* = 17.4, 10.8 Hz). Detailed analysis of its ^13^C NMR and DEPT spectra revealed 20 carbons resonance which were assigned as five methyls, five methylenes (one olefinic at *δ*_C_ 112.5), five methines (two olefinic at *δ*_C_ 127.1, 146.1) and five quaternary carbons (one olefinic at *δ*_C_ 175.3 and one carbonyl at *δ*_C_ 202.6). The ^1^H and ^13^C NMR of **2** were highly analogous to **5** [16], except for the following difference: in the ^13^C NMR spectrum, the downfield of **2** in chemical shifts of C-3 (*δ*_C_ 127.1) and C-4 (*δ*_C_ 175.3), together with one methylene in **5** was replaced with one carbonyl (*δ*_C_ 202.8). This might be caused by an additional carbonyl at C-2. In the HMBC spectrum, correlations could be observed from *δ*_H_ 5.69 (H-3) to *δ*_C_ 202.8 (C-2), *δ*_C_ 175.3 (C-4) and from *δ*_H_ 1.85 (H-10) to *δ*_C_ 202.8 (C-2), *δ*_C_ 175.3 (C-4). Thus, the carbonyl group was ascertained to be at C-2 position.

**Fig. 3:**
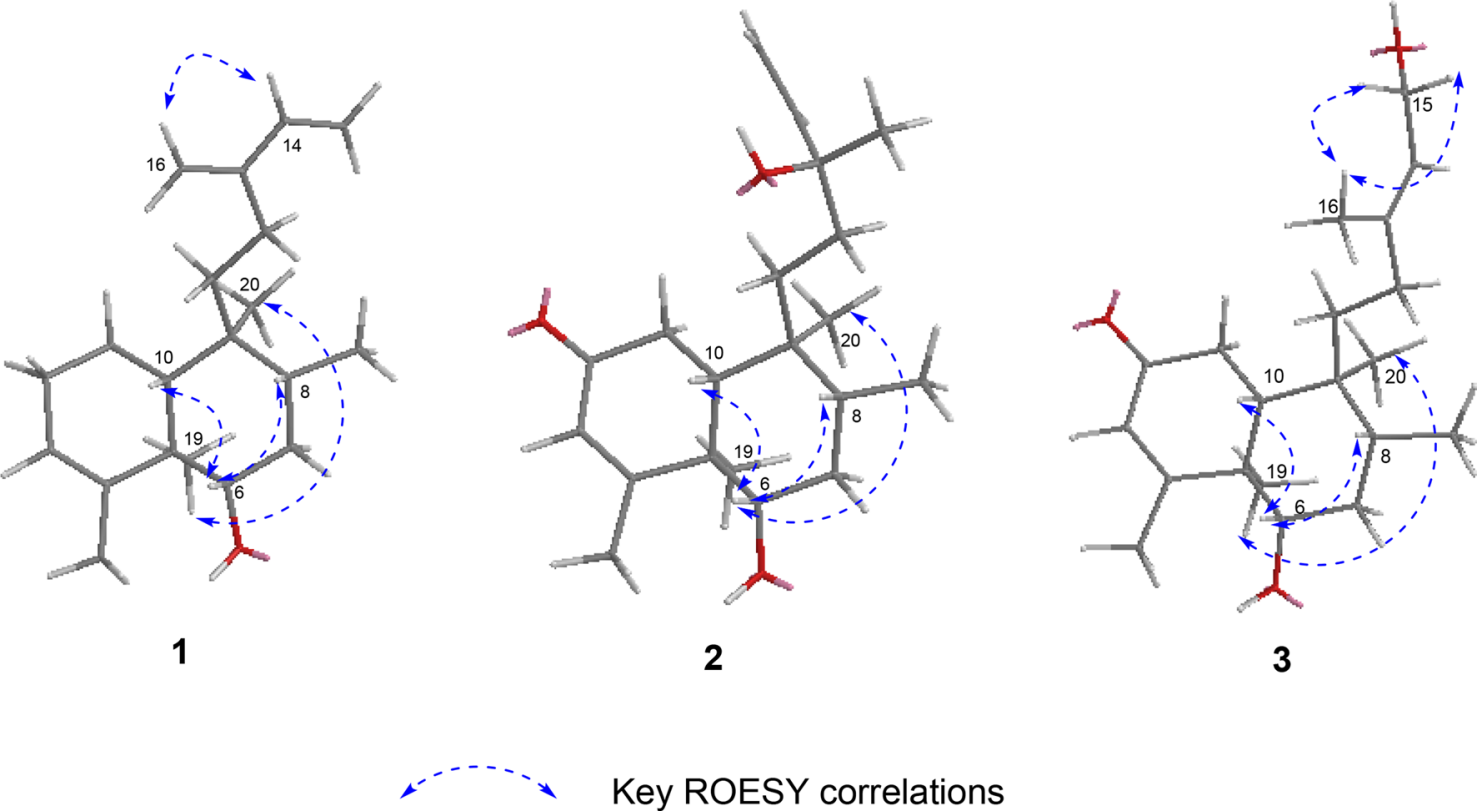
Key ROESY correlations of aglycones of compounds **1**–**3**

**Fig. 4:**
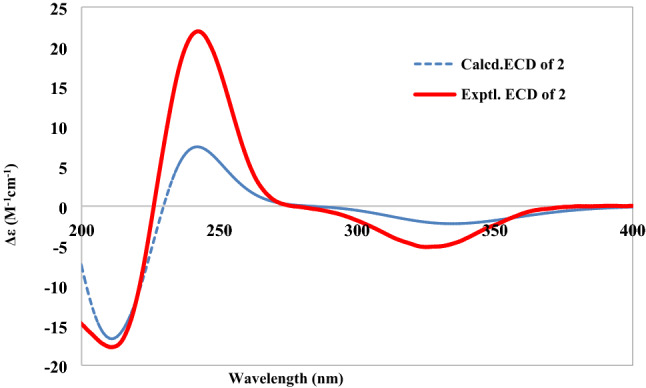
The experimental and calculated ECD spectra of compound **2**

The presence of *α*-l-rhamnose and *β*-d-glucose were identified by the same method as compound **1**. By the HMBC correlations of C-6 (*δ*_C_ 85)/H-1ʹ (*δ*_H_ 4.86) and C-4ʹ (*δ*_C_ 83.3)/H-1ʺ (*δ*_H_ 4.86, d, *J* = 7.8)(Fig. 2), the linkage positions of the sugar moieties were confirmed. The relative configurations of the diterpene were determined the same for their similar ROESY correlations as **1**: H-6/H-10, H-6/H-8, and CH_3_-19/CH_3_-20 (Fig. 3). Furthermore, the absolute configuration of **2** was determined by electronic circular dichroism (ECD) experiment which fitted well with the calculated ECD (Fig. 4). Finally, the structure of **2** was determined as (5*R*,6*S*,8*R*,9*S*,10*R*,13*S*)-6-*O*-[*β-*D-glucopyranosyl-(1 → 4)-*α*-l-rhamnopyranosyl]-2-ox-oneocleroda-3,13-dien-15-ol.

Compound **3** was obtained as pale yellow powder. It showed a molecular ion peak at *m/z* 651.3356 [M + Na]^+^ (calcd for 651.3351), corresponding to the molecular formula of C_32_H_52_O_12_, which indicating seven degrees of unsaturation. The 1D NMR spectra demonstrated two hexoses and a diterpene. The spectroscopic data of the sugar moieties were consistent with **1** and **2**, suggesting the presence of *α-*l-rhamnopyranosyl moiety and *β*-d-glucopyranosyl moieties. The diterpene part involved five methyls (one at *δ*_C_ 23.8), five methylenes (one oxygenated at *δ*_C_ 59.1), five methane (one olefinic at *δ*_C_ 125.5) and five quaternary carbons (one olefinic at *δ*_C_ 140.2). Those spectra suggested that the structure of **3** was highly similar to that of **2**. In comparison with **2**, the chemical shifts of C-14, C-15 and C-16 decreased from *δ*_C_ 146.1, 112.5 and 27.7 to *δ*_C_ 125.5, 59.1 and 23.8 respectively, while C-13 increased from *δ*_C_ 73.8 to 140.2, which testified the position of the double bond of **3** at C-13 and C-14. This was demonstrated again by the HMBC correlations of H_2_-15/C-13, C-14 and CH_3_-16/C-13, C-14. In the meantime, C-15 was determined to be a terminal oxymethylene by the chemical shift (*δ*_C_ 59.1) and ^1^H-^1^H COSY correlation of H_2_-15/H-14. Besides, the linkage of the sugar moieties was determined by the identical way with **1** and **2**. Thus the plannar structure of compound **3** was determined.

The relative configuration of **3** was assigned by ROESY correlations of H-6/H-10, H-6/H-8, and CH_3_-19/CH_3_-20. The ROESY correlations of CH_3_-16/H_2_-15 established the *E* configuration of the C-13/C-14 double bond. The experimental 1D and 2D NMR results of **2** and **3** were similar, which suggesting that they had the same (*R*,*S*,*R*,*S*,*R*) absolute configuration at C-5/6/8/9/10, respectively. Finally, the structure of **3** was determined as (5*R*,6*S*,8*R*,9*S*,10*R*)-6-*O*-[*β*-D-glucopyranosyl-(1 → 4)-*α*-l-rhamnopyranosyl]-(13*E*)-2-oxoneocleroda-3,14-dien-13-ol.

Additionally, the cytotoxicities of compounds **1**–**3** were evaluated against five human tumor cell lines HL-60, SMMC-7721, A-549, MCF-7 and SW-480, with cisplatin as the positive control. Compound **1** (40 μM) showed weak inhibitory activities in vitro against SMMC-7721, MCF-7 and SW480 with the inhibition ratio of 48.1%, 48.8% and 39.0%, respectively. However compounds **2** and **3** did not display any activities against the tested cell lines.

## Conclusion

In conclusion, this research led to the isolation of five compounds including three new and two known clerodane-type diterpene glycosides from *D. pedata*. Compounds **1**–**3** were assayed for their cytotoxicitives against five human tumor cell HL-60, SMMC-7721, A-549, MCF-7 and SW-480 with cisplatin as the positive control, in which compound **1** showed weak inhibitory activities in vitro against SMMC-7721, MCF-7 and SW480. This research enriched the chemical diversities of ferns.

## Experimental Section

### General Experimental Procedures

Optical rotations were carried out on Autopol VI automatoc polarimeter. UV spectra were obtained using a Shimadzu UV-2401 PC spectrophotometer. A Thermo Nicolet iS10 spectrometer was used for measuring IR spectroscopy, which used KBr pellets. 1D and 2D NMR spectra were recorded on Bruker DRX-600 and spectrometers with SiMe_4_ (TMS) as an internal standard. Chemical shifts (*δ*) are expressed in ppm with reference to the solvent signals. ESI and HRESIMS were performed on an UPLC-IT-TOF spectrometer. Semi-preparative HPLC was performed on an Agilent 1260 liquid chromatograph with a Zorbax SB-C18 (9.4 mm × 150 mm) column. Column chromatography was carried out using silica gel (100–200 mesh, Qingdao Haiyang Chemical Co. Ltd, Qingdao, People’s Republic of China) and macroporous absorption resin (DM 130, Tianjin Haoju Resin Technology Co. Ltd, people’s Republic of China). Fractions were monitored by TLC, and spots were visualized by heating silica gel plates sprayed with 10% H_2_SO_4_ in EtOH. The reagents used for acid hydrolysis and derivatization are listed: Trifluoroacetic acid (TFA, Beijing Yinuokai Technology Co, Ltd, Beijing, people’s Republic of China); 1-Phenyl-3-methyl-5-pyrazolon (PMP, analytical reagent, Tianjin Damao Chemical Reagent Factory); sodium dihydrogen phosphate (NaH_2_PO_4,_ analytical reagent, Tianjin Damao Chemical Reagent Factory, Tianjin, people’s Republic of China); d-glucose and l-rhamnose (Qingdao Jieshikang Biological Technology Co., Ltd, Qingdao, people’s Republic of China).

### Plant Material

The fronds of *Dicranopteris pedata* were collected from Huanglian Mountain, Lvchun County, Yunnan Province, People’s Republic of China, in September 2019, identified by Prof. Xiao Cheng (Kunming Institute of Botany, Chinese Academy of Sciences). The voucher specimen (No. 20190921) has been deposited in State Key Laboratory of Phytochemistry and Plant Resource in West China, Kunming Institute of Botany, the Chinese Academy of Sciences.

### Extraction and Isolation of Compounds 1–5

The dry fronds of *Dicranopteris pedata* (9 kg) were powdered and extracted with acetone (3 × 25 L) for 24 h at room temperature. The acetone extract was concentrated under reduced pressure to yield the residue (700 g), which was then suspended in H_2_O and subjected to column chromatography over DM 130 eluting with EtOH-H_2_O (0:10, 5:5, 7:3, 5:95, v/v), EtOH/H_2_O (5:5, v/v) fraction (230 g) was subjected to silica gel chromatography (CC) using silica gel (200–300 mesh) eluting with CHCl_3_-MeOH (from 12:1 to 0:1) to yield fractions A-D. Fraction B was subsequently separated on a Sephadex LH-20 CC (MeOH) to give two subfraction Fr.B_1_ (27 g) and Fr.B_2_ (25 g). Fr.B_1_ was divided into ten fractions (from Fr.B_1_-1 to Fr.B_1_-10) by using RP-C18 eluting with MeOH-H_2_O (from 1:9 to 1:1). Fr.B_1_-10 was separated by CC on silica gel (CHCl_3_-MeOH, 10:1) and further purified by semi-preparative HPLC with MeCN-H_2_O (23:77, v/v) to obtain compound **2** (6 mg, *t*_R_ = 44.11 min). Compound **4** (4 mg, *t*_R_ = 26.70 min) and **5** (3 mg, *t*_R_ = 31.35 min) were obtained by MeOH-H_2_O (40:60, v/v). Compound **3** was separated and purified from Fr.B_1_-8 by semi-preparative HPLC with MeOH-H_2_O (48:52, v/v). Fr.B_1_-9 was separated by CC on silica gel (CHCl_3_-MeOH, 20:1 to 15:1) and further purified by semi-preparative HPLC with MeOH-H_2_O (75:25, v/v) to give compound **1** (4 mg, *t*_R_ = 36.11 min).

### Spectroscopy Data of Compounds 1–3

(5*R*,6*S*,8*R*,9*S*,10*R*)-6-*O*-[*β*-d-glucopyranosyl-(1 → 4)-*α*-l-rhamnopyranosyl]cleroda-3,13(16),14-diene (**1**): pale yellow powder; [*α*]_D_^26^ − 25.8 (*c*, 0.26, MeOH); UV (MeOH) λ_max_ (log ε): 196.5 (3.26), 208.0 (3.17), 223.5 (3.27); IR (*ν*_max_): 3402, 3090, 2956, 2926, 2875 cm^−1^; HRESIMS at *m*/*z* 619.3454 [M + Na]^+^ (calcd for 619.3458). ^1^H and ^13^C NMR data, see Tables 1 and 2.

**Table 1:** ^1^H NMR (600 Hz) data (*δ* in ppm, *J* in Hz) for compounds **1**–**3** in CD_3_OD

NO.	1	2	3
	*δ*_H_, mult (*J*)	*δ*_H_, mult (*J*)	*δ*_H_, mult (*J*)
1	1.61 (overlapped)	*β,* 2.51, dd (17.9, 13.9)	*β,* 2.59, dd (17.8, 14.0)
1		*α*, 2.28, dd (17.9, 3.2)	*α,* 2.33, dd (17.8, 2.9)
2	2.03 (overlapped)		
3	5.24, br s	5.69, s	5.71, s
6	3.43, dd (11.2, 4.7)	3.64 (overlapped)	3.68 (overlapped)
7	*β*, 2.03 (overlapped)	*β*, 2.12, m	*β*, 2.16, dd (10.4, 4.6)
	*α*, 1.59 (overlapped)	*α*, 1.68, m	*α*, 1.71 (overlapped)
8	1.70 (overlapped)	1.62, m	1.73 (overlapped)
10	1.42 (overlapped)	1.85, dd (13.9, 3.4)	1.95 (overlapped)
11	*β*, 1.54, m	*β*, 1.36 (overlapped)	1.35 (overlapped)
	*α*, 1.44 (overlapped)	*α*, 1.27 (0verlapped)	
12	*β*, 2.10, td (13.2, 4.1)	*β*, 1.37 (overlapped)	*β*, 1.95 (overlapped)
	*α*, 1.98 (m)	*α*, 1.17 (overlapped)	*α*, 1.74 (m)
14	6.37, dd (17.6,10.9)	5.84, dd (17.4, 10.8)	5.33, t (6.8)
15	*β*, 5.20, d (17.6)	*β*, 5.16, dd (17.4, 1.5)	4.0, d (6.9)
	*α*, 5.04, d (10.9)	*α*, 5.03, dd (10.8, 1.5)	
16	4.97, d (4.4)	1.22, s	1.72, s
17	0.84, d (6.7)	0.85 (overlapped)	0.89, d (6.1)
18	1.73, d (1.1)	2.06, d (2.06)	2.08, s
19	1.08, s	1.17, s	1.20, s
20	0.73, s	0.84 (overlapped)	0.84, s
Rha			
1*ʹ*	4.79, d (1.5)	4.86 (overlapped)	4.88 (overlapped)
2*ʹ*	3.84 (overlapped)	3.84 (overlapped)	3.85 (overlapped)
3*ʹ*	3.88, dd (9.4, 3.2)	3.88, dd (9.3, 3.2)	3.89, dd (9.3, 3.2)
4*ʹ*	3.62, t (9.5)	3.62, (overlapped)	3.65, t (9.7)
5*ʹ*	3.78, m	3.77, m	3.77, m
6*ʹ*	1.31, d (6.3)	1.32, d (6.2)	1.33, d (6.2)
Glc			
1ʺ	4.59, d (7.8)	4.59, d (7.8)	4.60, d (7.8)
2*ʺ*	3.20, dd (9.1, 7.9)	3.21, dd (9.1, 7.9)	3.21, m
3*ʺ*	3.36, dd (15.8, 6.9)	3.36, m	3.36, m
4*ʺ*	3.29 (overlapped)	3.29 (overlapped)	3.29 (overlapped)
5*ʺ*	3.26, m	3.26, m	3.26, m
6*ʺ*	*β*, 3.85 (overlapped)	*β*, 3.85 (overlapped)	*β*, 3.84 (overlapped)
	*α*, 3.69, dd (11.9, 5.2)	*α*, 3.69, dd (11.8, 5.2)	*α*, 3.69, dd (overlapped)

**Table 2:** ^13^C NMR (150 Hz) data (*δ* in ppm) for compounds **1**–**3** in CD_3_OD

NO	1		2		3	
	*δ*_C_	Type	*δ*_C_	Type	*δ*_C_	Type
1	18.9	CH_2_	35.0	CH_2_	35.2	CH_2_
2	27.7	CH_2_	202.8	C	202.6	C
3	123.6	CH	127.1	CH	127.1	CH
4	144.6	C	175.3	C	175.2	C
5	45.1	C	46.6	C	46.7	C
6	87.7	CH	85.0	CH	85.0	CH
7	36.1	CH_2_	35.6	CH_2_	35.5	CH_2_
8	35.4	CH	34.9	CH	34.9	CH
9	39.4	C	39.0	C	39.5	C
10	47.1	CH	46.4	CH	46.5	CH
11	39.1	CH_2_	32.4	CH_2_	37.3	CH_2_
12	25.5	CH_2_	35.5	CH_2_	25.8	CH_2_
13	148.9	C	73.8	C	140.2	C
14	140.1	CH	146.1	CH	125.5	CH
15	113.3	CH_2_	112.5	CH_2_	59.1	CH_2_
16	116.2	CH_2_	27.7	CH_3_	23.8	CH_2_
17	16.3	CH_3_	15.9	CH_3_	16.0	CH_3_
18	23.3	CH_3_	23.5	CH_3_	23.6	CH_3_
19	16.6	CH_3_	14.9	CH_3_	14.8	CH_3_
20	18.3	CH_3_	17.9	CH_3_	17.7	CH_3_
Rha						
1*ʹ*	103.2	CH	103.0	CH	103.0	CH
2*ʹ*	72.6	CH	72.5	CH	72.5	CH
3*ʹ*	72.7	CH	72.6	CH	72.6	CH
4*ʹ*	83.5	CH	83.3	CH	83.4	CH
5*ʹ*	68.8	CH	68.9	CH	69.0	CH
6*ʹ*	17.9	CH_3_	17.9	CH_3_	17.9	CH_3_
Glc						
1*ʺ*	105.7	CH	105.7	CH	105.7	CH
2*ʺ*	76.1	CH	76.1	CH	76.1	CH
3*ʺ*	78.1	CH	78.1	CH	78.1	CH
4*ʺ*	71.5	CH	71.5	CH	71.5	CH
5*ʺ*	78.2	CH	78.2	CH	78.2	CH
6*ʺ*	62.7	CH_2_	62.7	CH_2_	62.7	CH_2_

(5*R*,6*S*,8*R*,9*S*,10*R*,13*S*)-6-*O*-[*β-*d-glucopyranosyl-(1 → 4)-*α*-l-rhamnopyranosyl]-2-oxoneocleroda-3,13-dien-15-ol (**2**): pale yellow powder; [*α*]_26_^D^ − 37.98 (*c*, 0.10, MeOH); UV (MeOH) λ_max_ (log ε): 195.5 (3.13), 209.0 (3.02), 238.5 (3.35); ECD (MeOH) λ_max_ (Δε) 245 (21.24); IR (*ν*_max_): 3387, 2964, 2926, 2878, 1652 cm^−1^; HRESIMS at *m/z* 651.3355 [M + Na]^+^ (calcd for 651.3357); ^1^H and ^13^C NMR data see Tables 1 and 2.

(5*R*,6*S*,8*R*,9*S*,10*R*)-6-*O*-[*β*-d-glucopyranosyl-(1 → 4)-*α*-l-rhamnopyranosyl]-(13*E*)-2-oxoneocleroda-3,14-dien-13-ol (**3**): pale yellow powder; [*α*]_26_^D^ − 18.9 (*c*, 0.26, MeOH); UV (MeOH) λ_max_ (log ε): 196.0 (3.32), 215.0 (2.99), 238.0 (3.19); IR (*ν*_max_): 3404, 2964, 2928, 1716, 1651 cm^−1^; HRESIMS at *m/z* 651.3356 [M + Na]^+^ (calcd for 651.3351). ^1^H and ^13^C NMR data see Tables 1 and 2.

### Acid Hydrolysis and Derivatization

Compounds **1**–**3** were hydrolyzed with trifluoroacetic acid (TFA) by the procedure of the previous reported [17], with minor modifications. Compound **1** (2 mg, 3.35 μmol) was dissolved in 2 M TFA (2 mL), after the reaction mixture was heated to 120 °C and stirred for 2 h before cooling to room temperature. Then, extracted with CHCl_3_ (3 × 1 mL) and the aqueous layer was concentrated for next derivatization. Next, adding 0.3 M NaOH (1 mL) and 0.5 M PMP (1 mL) to the aqueous layer. The reaction was heated to 75 °C and stirred for 1.5 h before cooling to room temperature. Then the reaction mixture was quenched with 0.3 M HCl (1 mL) and extracted with CHCl_3_ (3 × 2 mL). Further analysis the aqueous layer over HPLC [*t*_R_ = 6.67, 12.78 min; 40% MeOH: 60% sodium phosphate (pH 6.8); 1.5 mL/min]. The acid hydrolysis and HPLC analysis of **2** and **3** were manipulated by the same way as that of **1**. Likewise, the standard monosaccharides, d-Glc (2 mg) and l-Rha (2 mg) were derivatized with PMP as compounds **1**–**3**, then HPLC analysis in the same condition as **1**–**3**. The sugar moieties of compounds **1**–**3** were identified as d-Glc (*t*_R_ = 12.78 min) and l-Rha (*t*_R_ = 6.67 min), showing retention times consistent with the standard monosaccharide derivatives.

### Cytotoxicity Assay

MTT assays [18] were used to evaluating the cytotoxicities of compounds **1**–**3**. Five human tumor cell lines including leukemia HL-60, liver cancer SMMC-7721, lung cancer A-549, breast cancer MCF-7 and colon cancer SW-480, were incubated in RMPI-1640 or DMEM medium supplemented with 10% fetal bovine serum and seeded at 3–15 × 10^3^ cells each well of a 96-well cell culture plate. After 12–24 h of incubation at 37 °C, the tested compound (40 μM, dissolved in DMSO) was added. After incubated for 48 h, each well were added 20 μL MTS [3-(4,5-dimethylthiazol-2-yl)-5-(3-carboxymethoxyphenyl)-2-(4-sulfophenyl)-2H-tetrazolium, inner salt]. Then, they were cultured for further 4 h. The cisplatin was applied as the positive control. The MULTISKAN FC was used to measure the OD value at 492 nm.

## Supplementary Information

Below is the link to the electronic supplementary material.

Supplementary file1 (DOCX 1800 kb)

## Data Availability

This article is licensed under a Creative Commons Attribution 4.0 International License, which permits to use, sharing, adaptation, distribution and reproduction in any medium or format, as long as you give appropriate credit to the original author (s) and the source, provide a link to the Creative Commons licence, and indicate if changes were made. The images or other third party material in this article are included in the article’s Creative Commons licence, unless indicated otherwise in a credit line to the material. If material is not included in the article’s Creative Commons licence and your intended use is not permitted by statutory regulation or exceeds the permitted use, you will need to obtain permission directly from the copyright holder. To view a copy of this licence, visit http://creativecommons.org/licenses/by/4.0/.
