# Supplementary material for: Clerodane-type Diterpene Glycosides from Dicranopteris pedata
Source: Nat Prod Bioprospect. 2021 Jun 5;11(5):557–64. doi: 10.1007/s13659-021-00315-y (PMC8390628; doi:10.1007/s13659-021-00315-y)
Supplement: Supplementary file 1 — Supplementary file1 (DOCX 1800 kb) [file 13659_2021_315_MOESM1_ESM.docx]

**Clerodane-type Diterpene Glycosides from *Dicranopteris pedata***

Bei-Bei Gao^2,3^, Yu-Fei Ou^1,2,3^, Qin-Feng Zhu^4^, Zhi-Ping Zhou^1,2^, Zhen-Tao Deng^1,2^, Meng Li^1,2^, Qin-Shi Zhao^1*^

*^1^ Key Laboratory of Medicinal Chemistry for Natural Resource, Ministry of Education, School of Chemical Science and Technology, Yunnan University, Kunming, Yunnan 650091, P. R. China*

*^2^ State Key Laboratory of Phytochemistry and Plant Resources in West China and Yunnan Key Laboratory of Natural Medicinal Chemistry, Kunming Institute of Botany, Chinese Academy of Sciences, Kunming 650201, China*

*^3^ Bei-Bei Gao and Yu-Fei Ou have contributed equally to this work.*

*^4^ Guizhou Medical University, College of Pharmacy, Guian New Area, 550025, Guizhou, China*

*^* Corresponding author.^*

*^E-mail address:^* ^Qin-shi Zhao:^ [^qinshizhao@mail.kib.ac.cn^](mailto:qinshizhao@mail.kib.ac.cn)

Supplementary data

**Figure S1-S8 The original NMR and MS spectra of compound 1**

**Figure S9-S16. The original NMR and MS spectra of compound 2**

**Figure S17-S24. The original NMR and MS spectra of compound 3**

**Figure S25. The retention time of Rha and Glc derivatives in HPLC (DAD, sig = 254nm)**


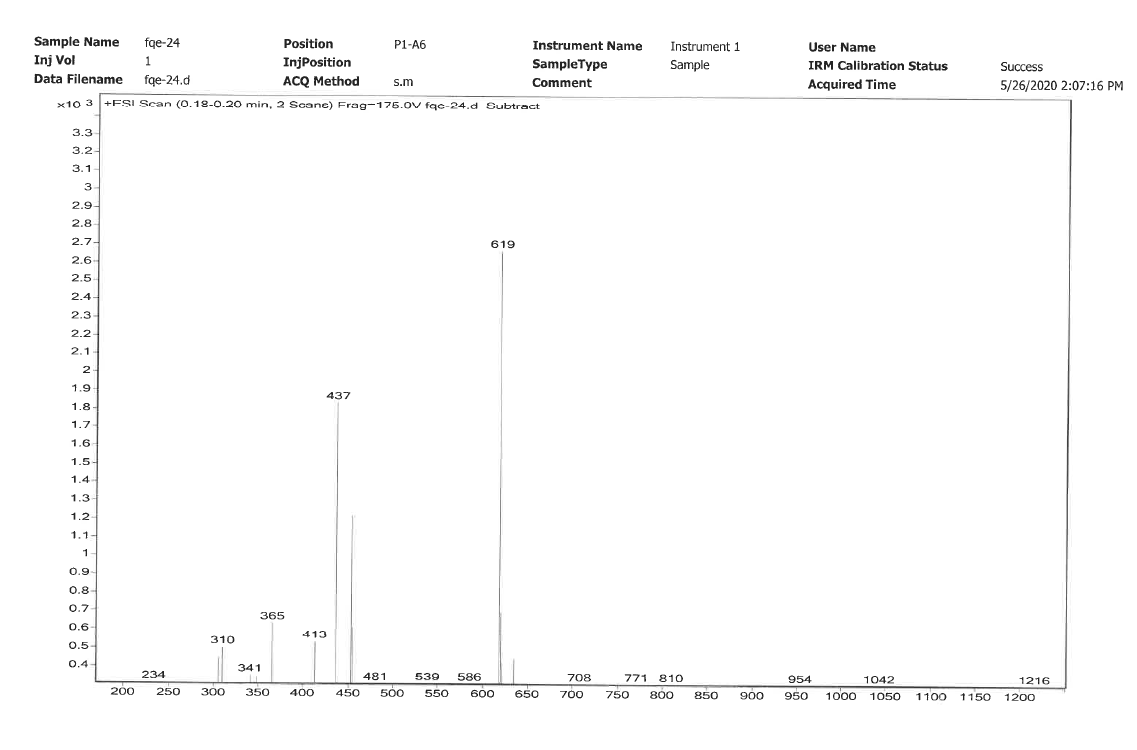


**Figure S1**. ESI spectrum of compound **1**, in positive ion mode


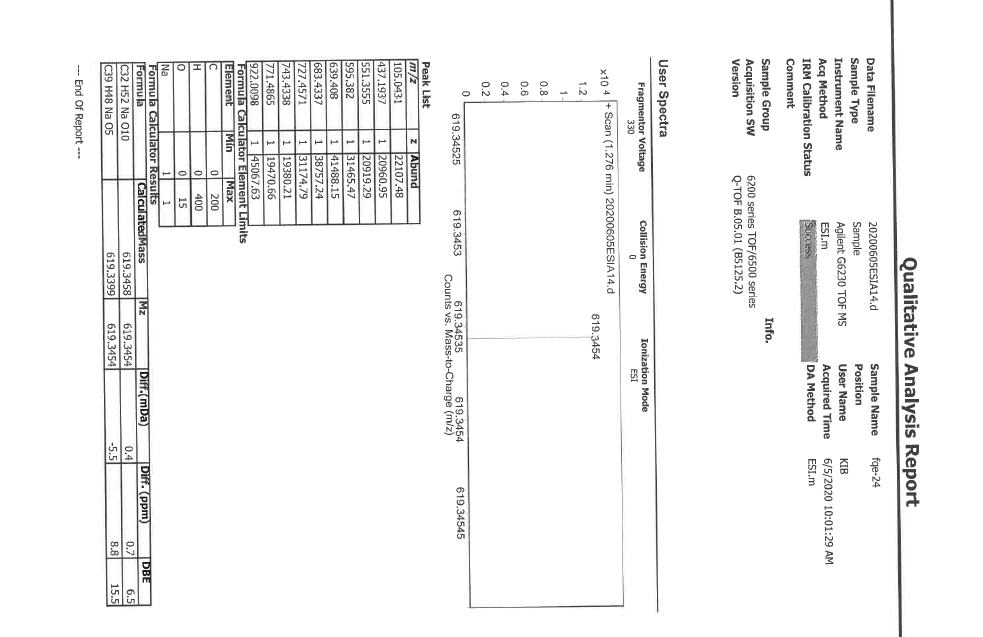


**Figure S2**. HRMSESI spectrum of compound **1**, in positive ion mode


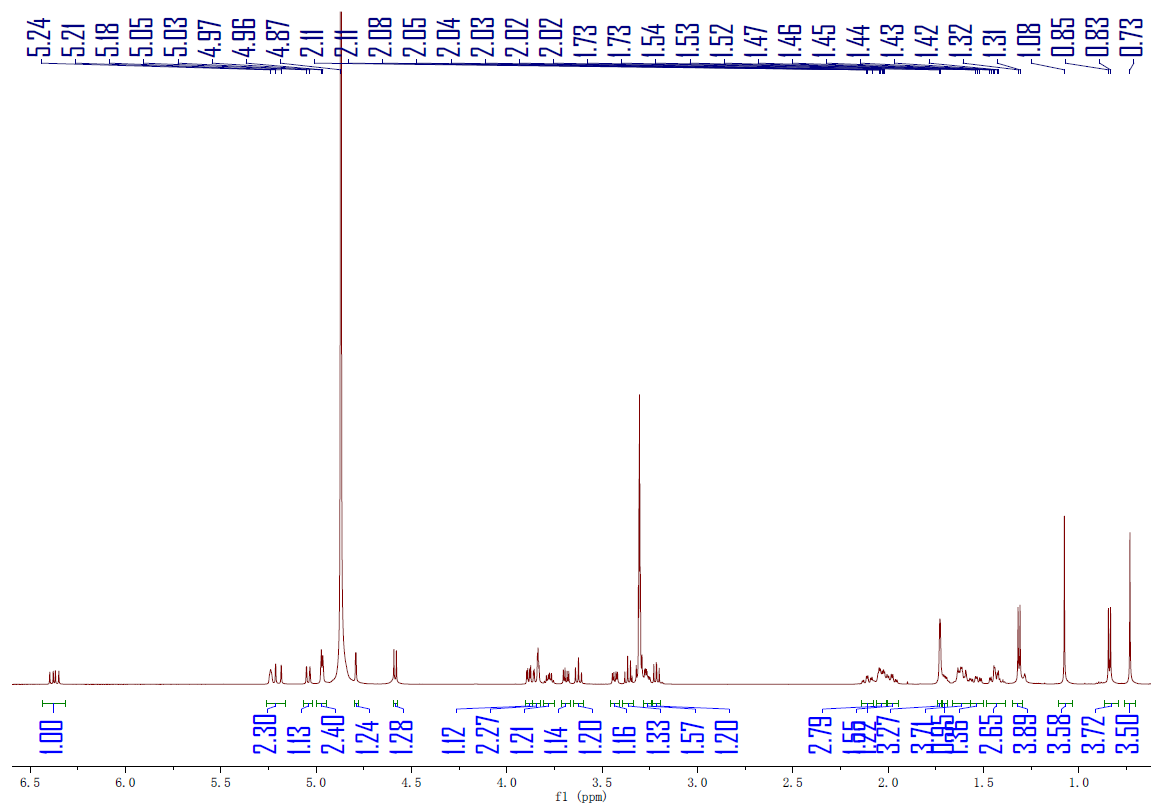


**Figure S3**. ^1^H NMR Spectrum (600 MHz, CD_3_OD) of compound **1**


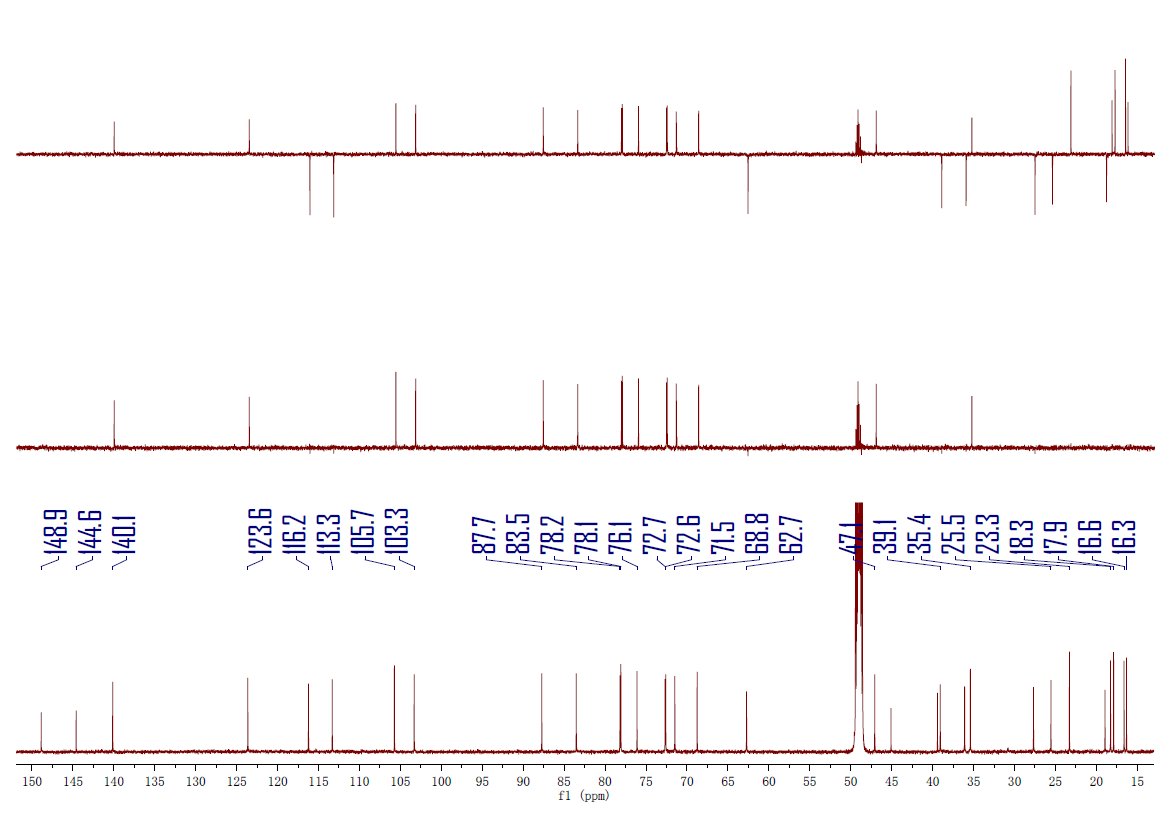


**Figure.S4**. ^13^C NMR Spectrum (150MHz, CD_3_OD) of compound **1**


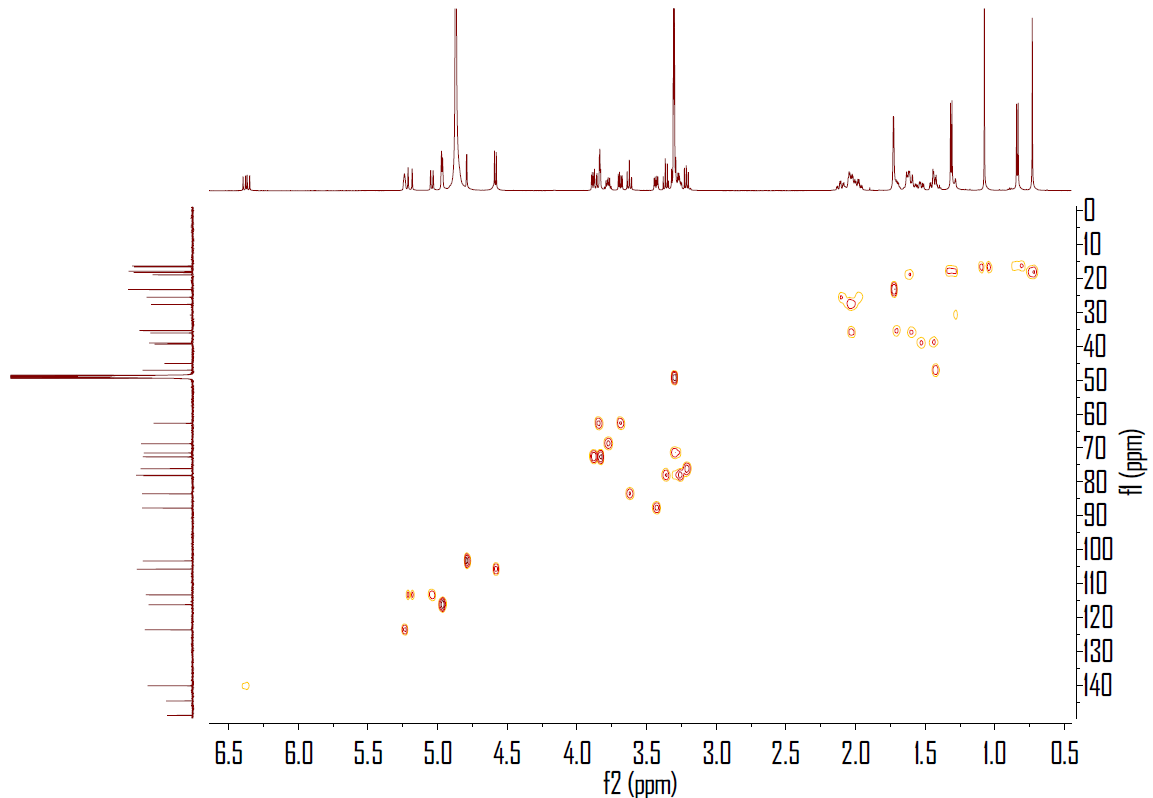


**Figure.S5**. HSQC Spectrum (CD_3_OD) of compound **1**


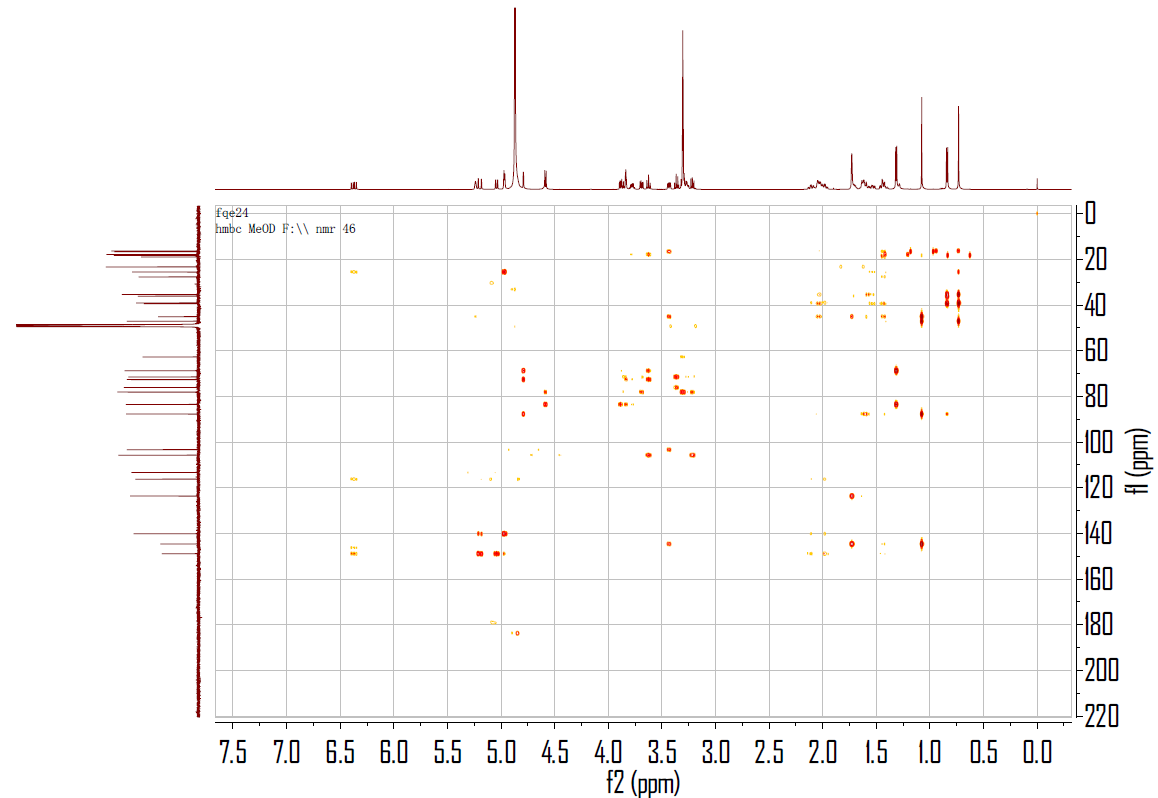


**Figure.S6**. HMBC Spectrum (CD_3_OD) of compound **1**


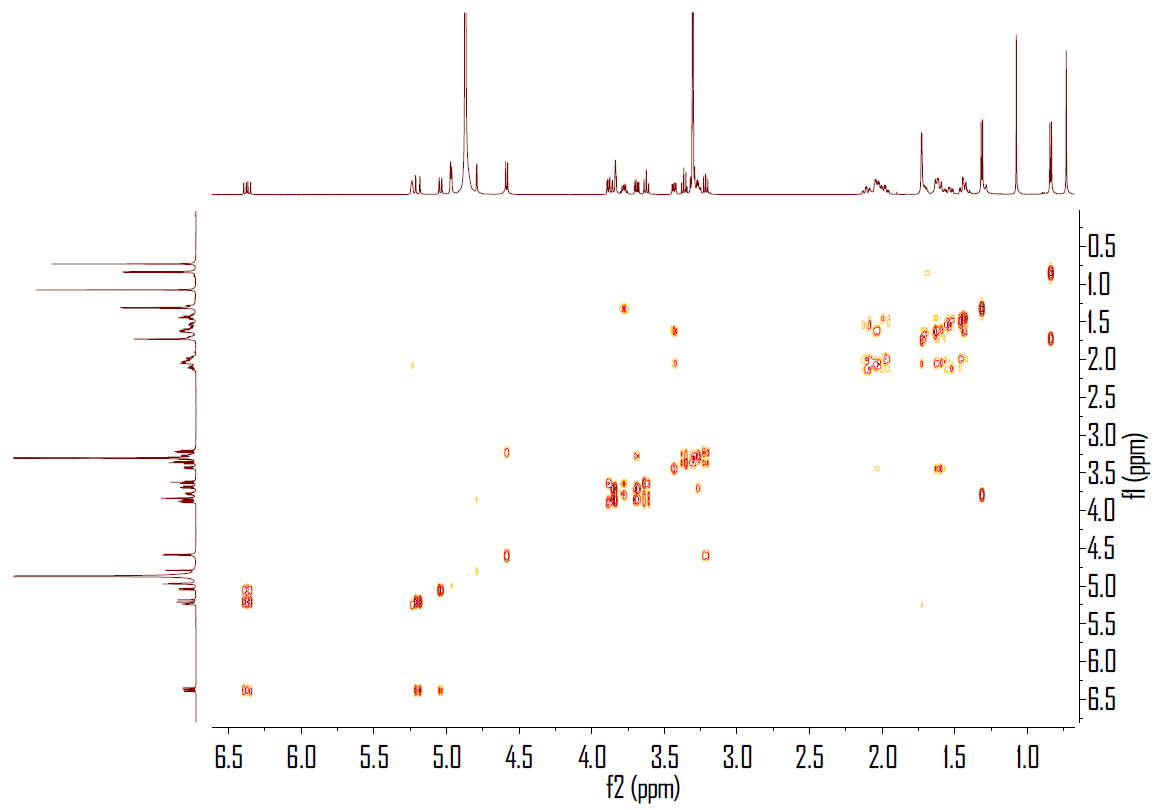


**Figure.S7**. COSY Spectrum (CD_3_OD) of compound **1**


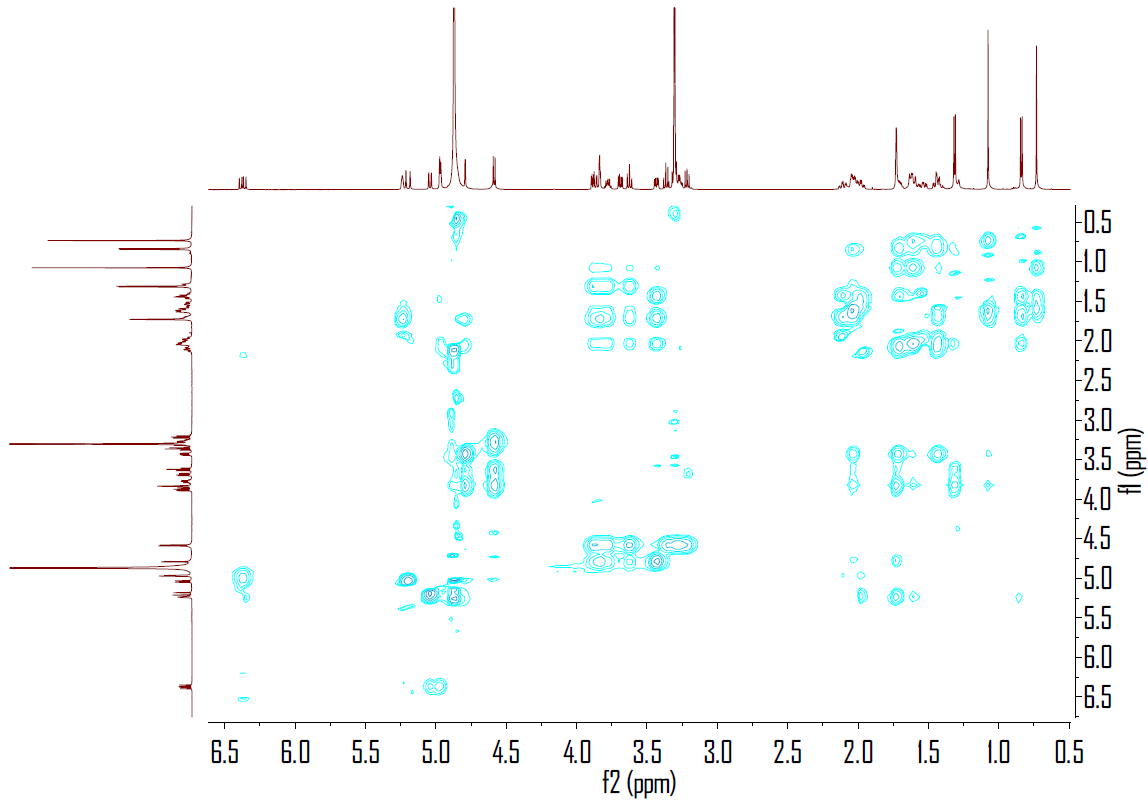


**Figure S8**. ROESY Spectrum (CD_3_OD) of compound **1**


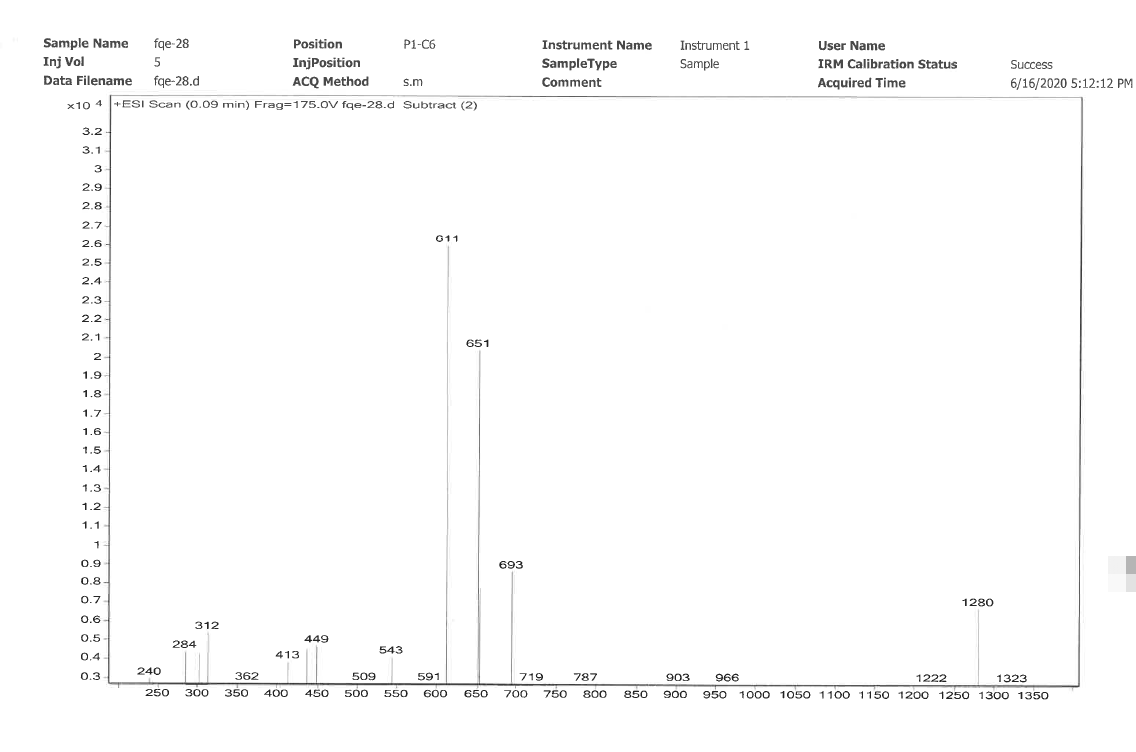


**Figure S9**. ESI spectrum of compound **2**, in positive ion mode


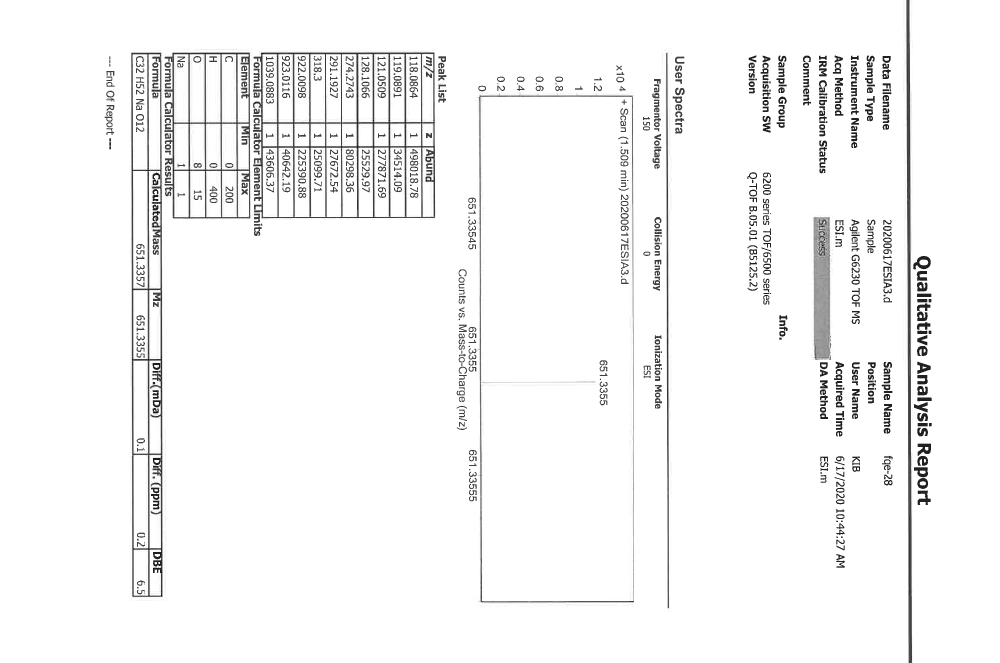


**Figure S10**. HRMSESI spectrum of compound **2**, in positive ion mode


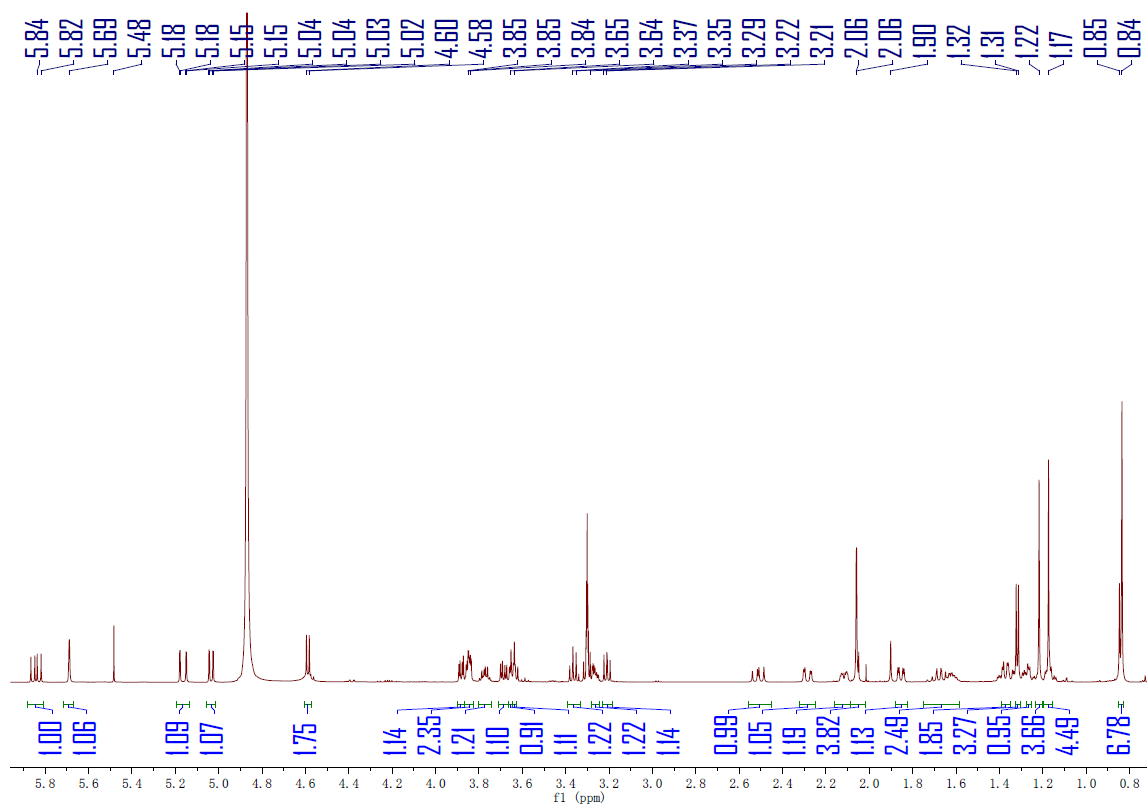


**Figure S11**. ^1^H NMR Spectrum (600 MHz, CD_3_OD) of compound **2**


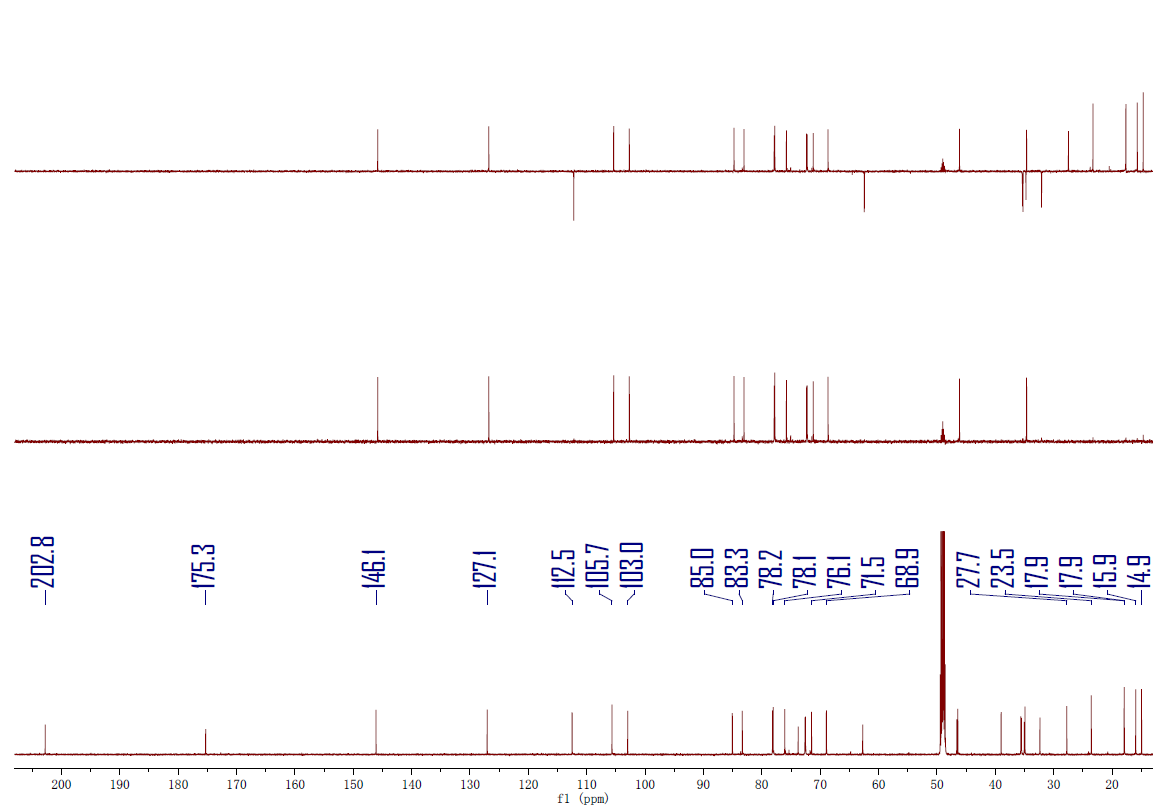


**Figure.S12**. ^13^C NMR Spectrum (150MHz, CD_3_OD) of compound **2**


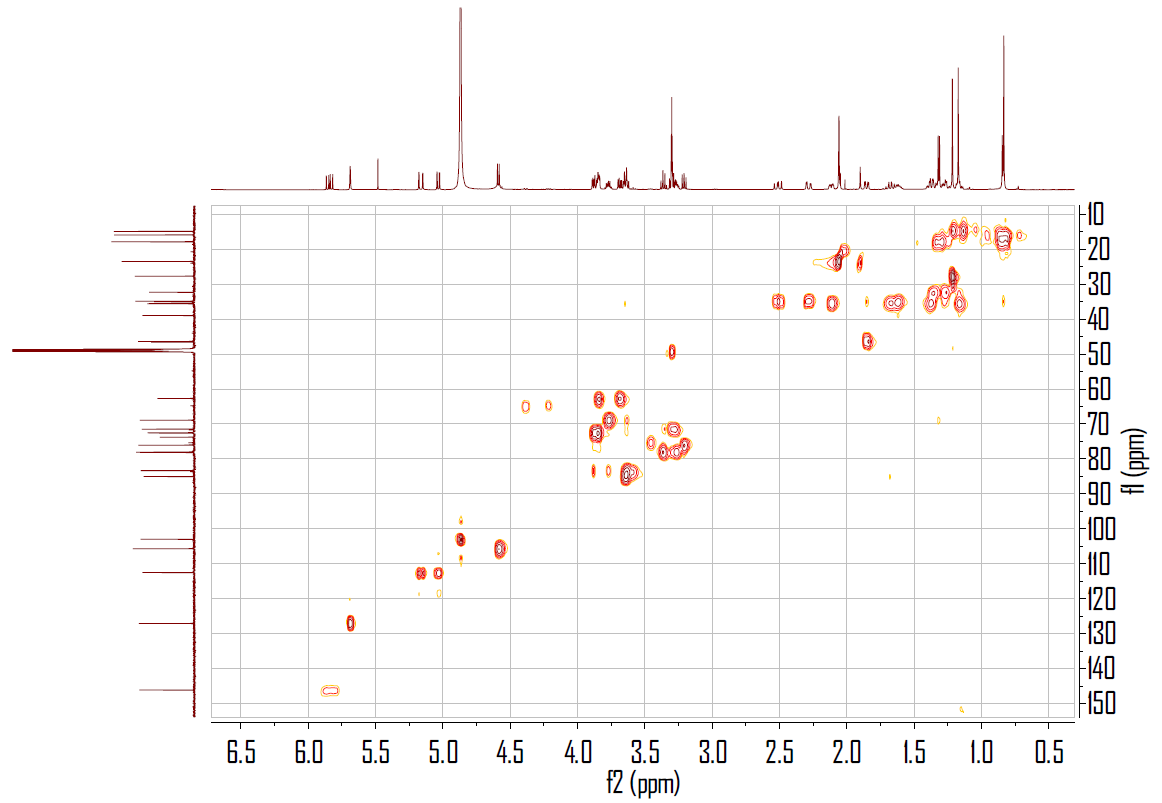


**Figure.S13**. HSQC Spectrum (CD_3_OD) of compound **2**


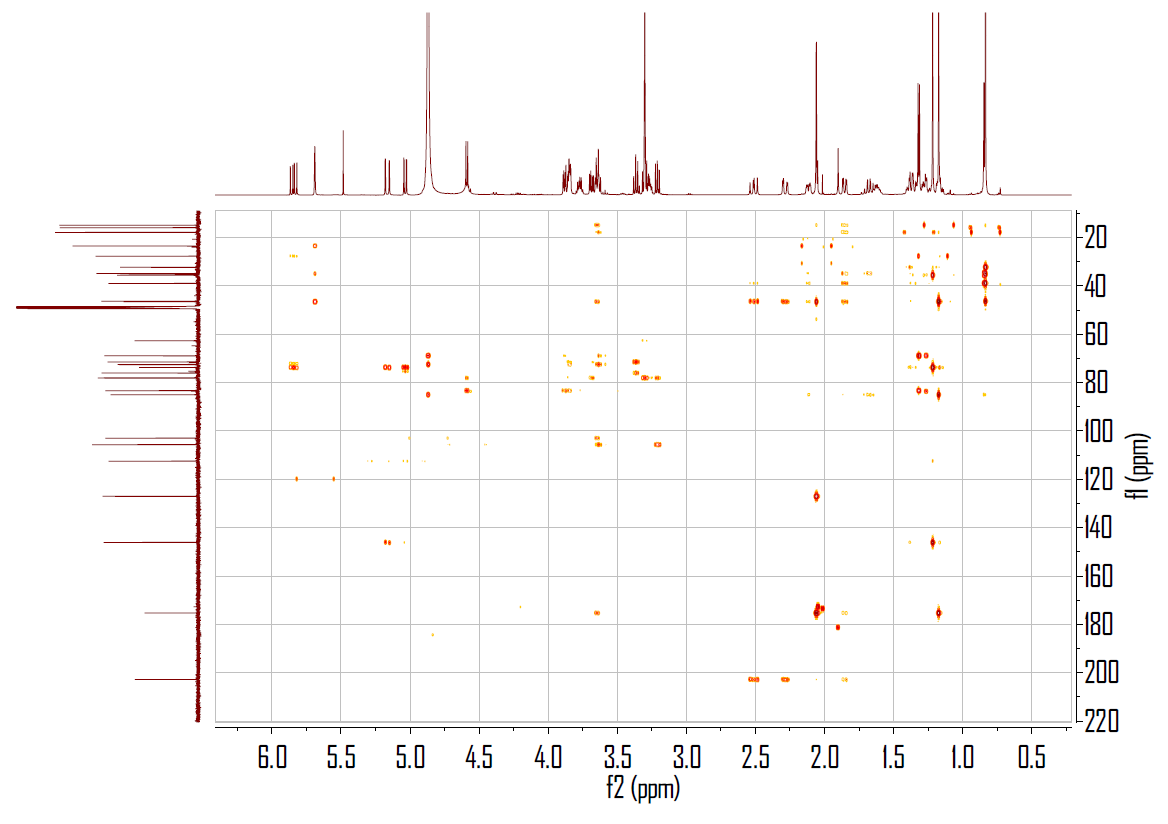


**Figure.S14**. HMBC Spectrum (CD_3_OD) of compound **2**


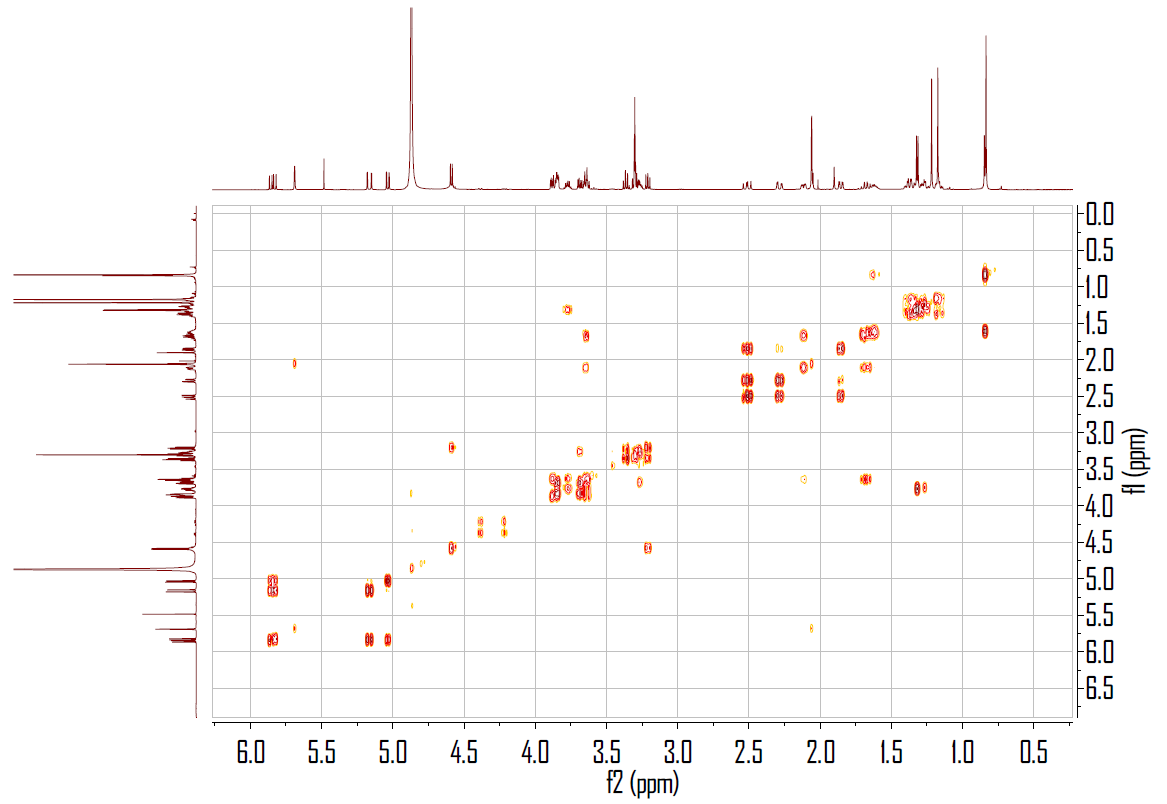


**Figure.S15**. COSY Spectrum (CD_3_OD) of compound **2**


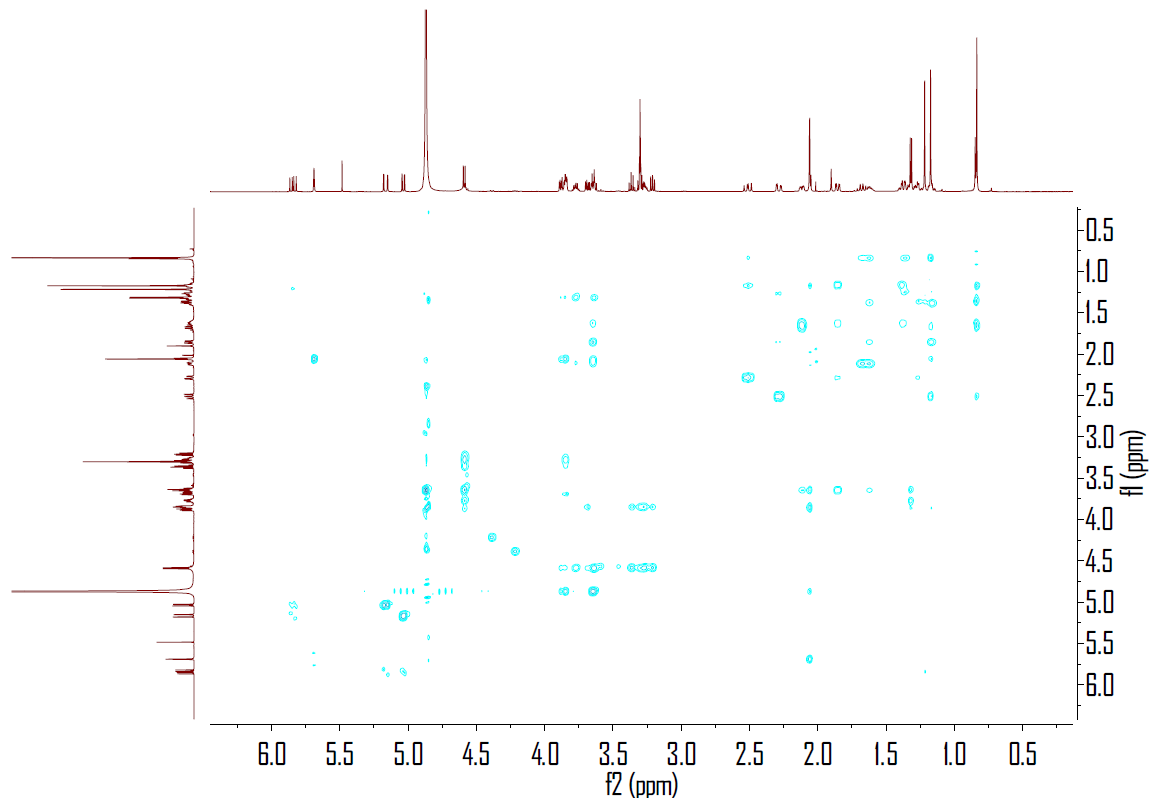


**Figure S16**. ROESY Spectrum (CD_3_OD) of compound **2**


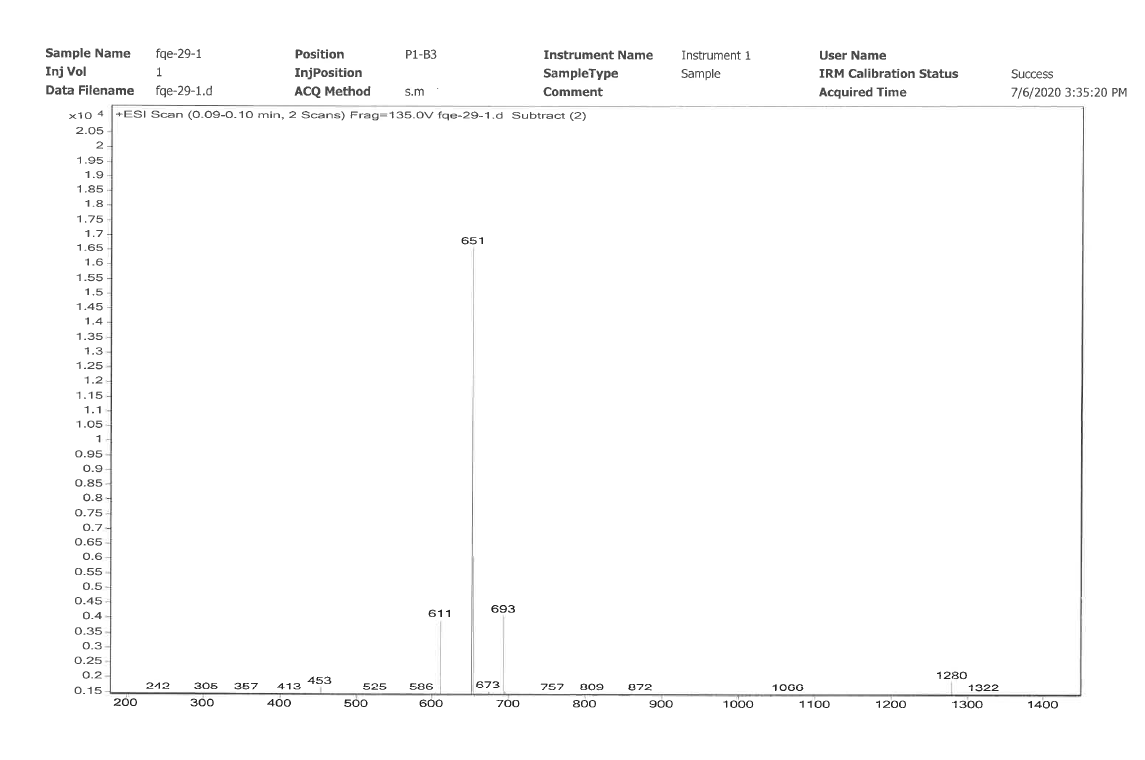
**Figure S17**. ESI spectrum of compound **3**, in positive ion mode


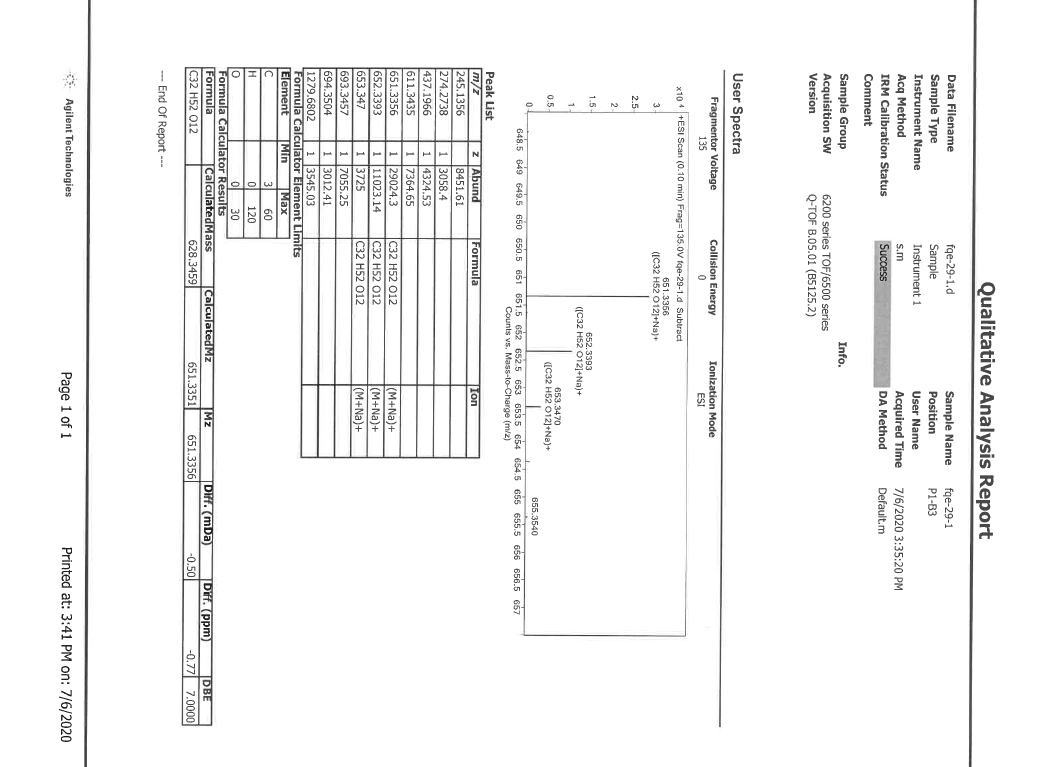


**Figure S18**. HRMSESI spectrum of compound **3**, in positive ion mode


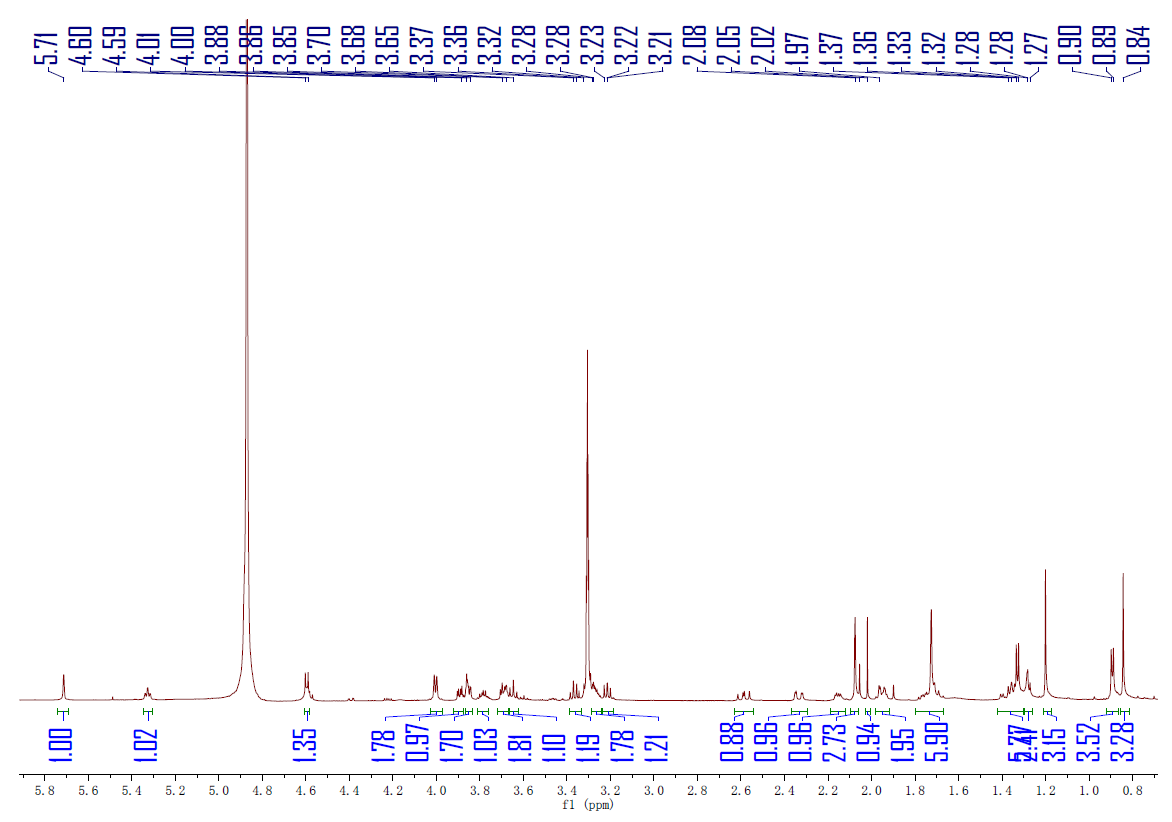


**Figure S19**. ^1^H NMR Spectrum (600 MHz, CD_3_OD) of compound **3**


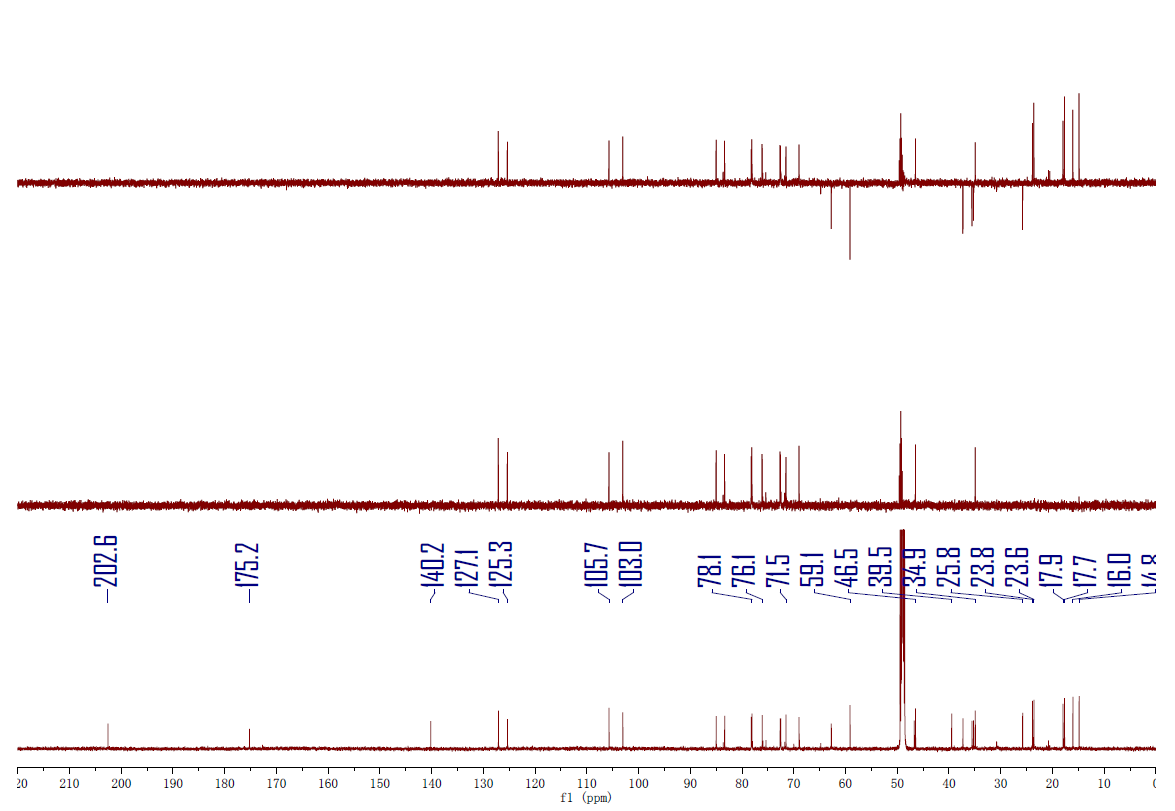


**Figure.S20**. ^13^C NMR Spectrum (150MHz, CD_3_OD) of compound **3**


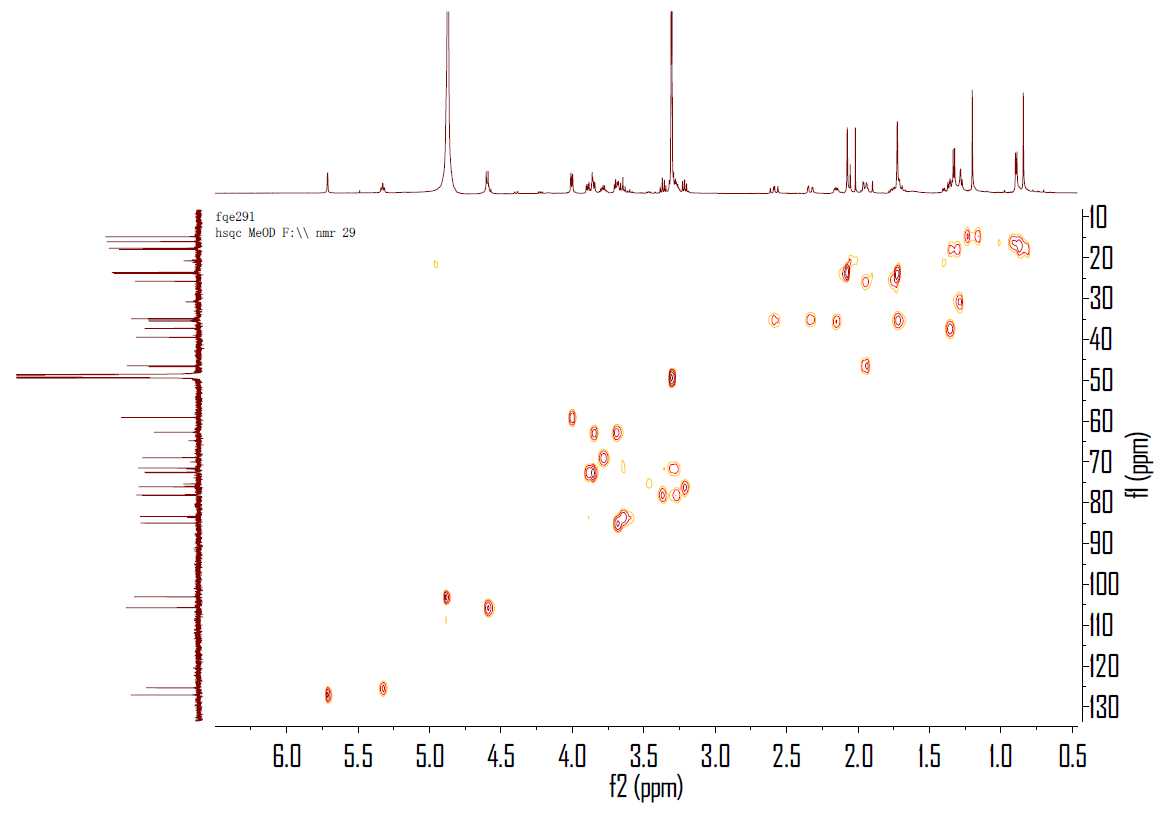


**Figure.S21**. HSQC Spectrum (CD_3_OD) of compound **3**


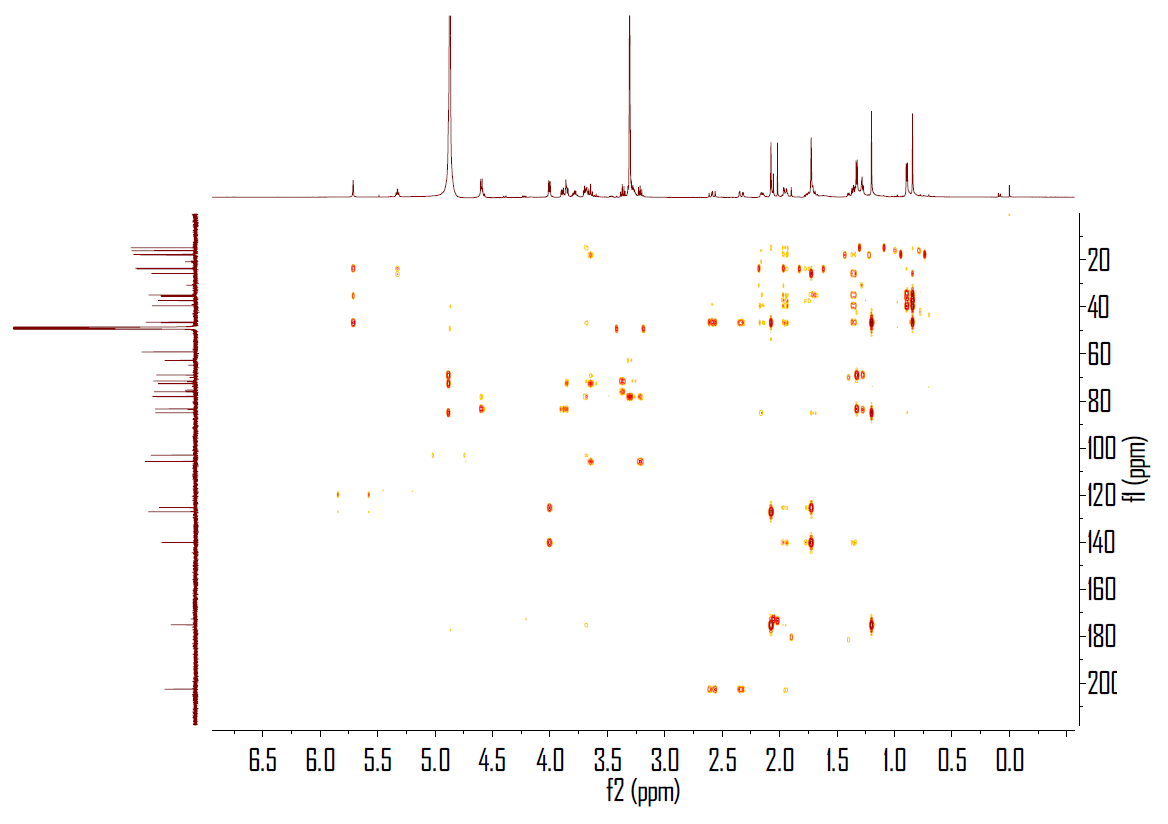


**Figure.S22**. HMBC Spectrum (CD_3_OD) of compound **3**


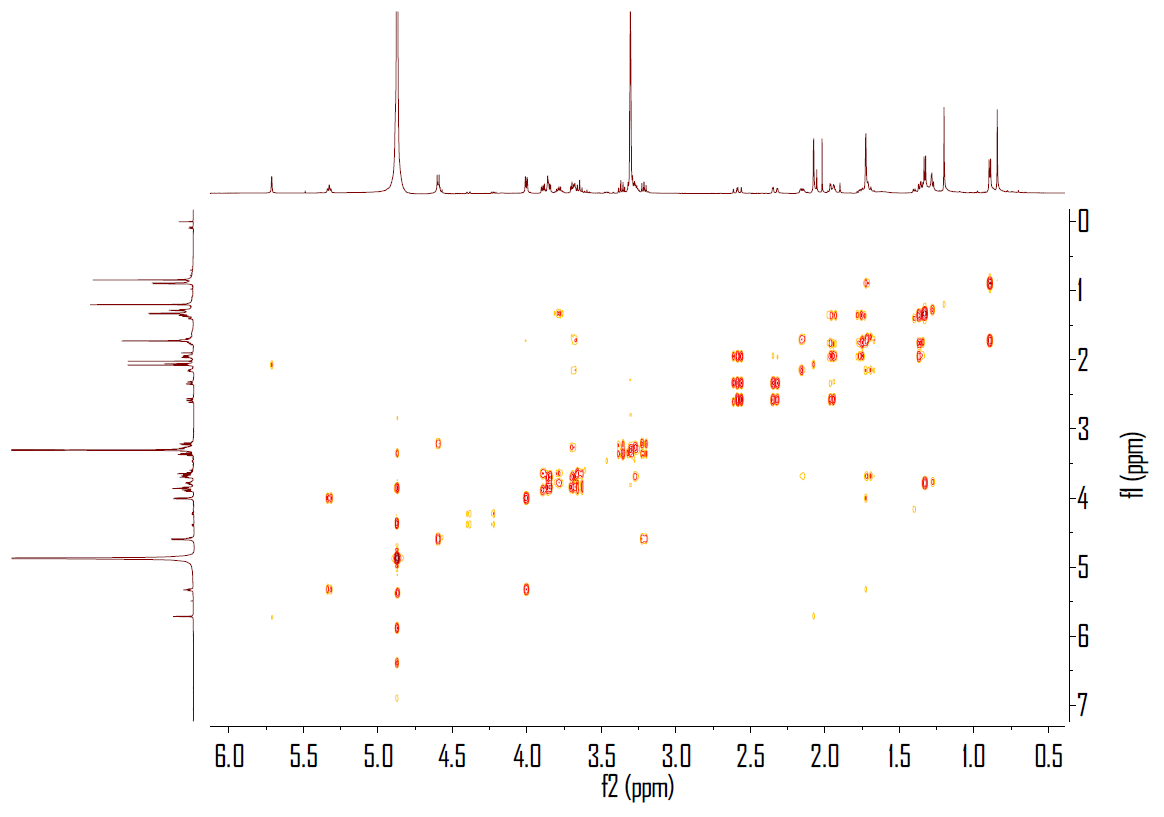


**Figure.S23**. COSY Spectrum (CD_3_OD) of compound **3**


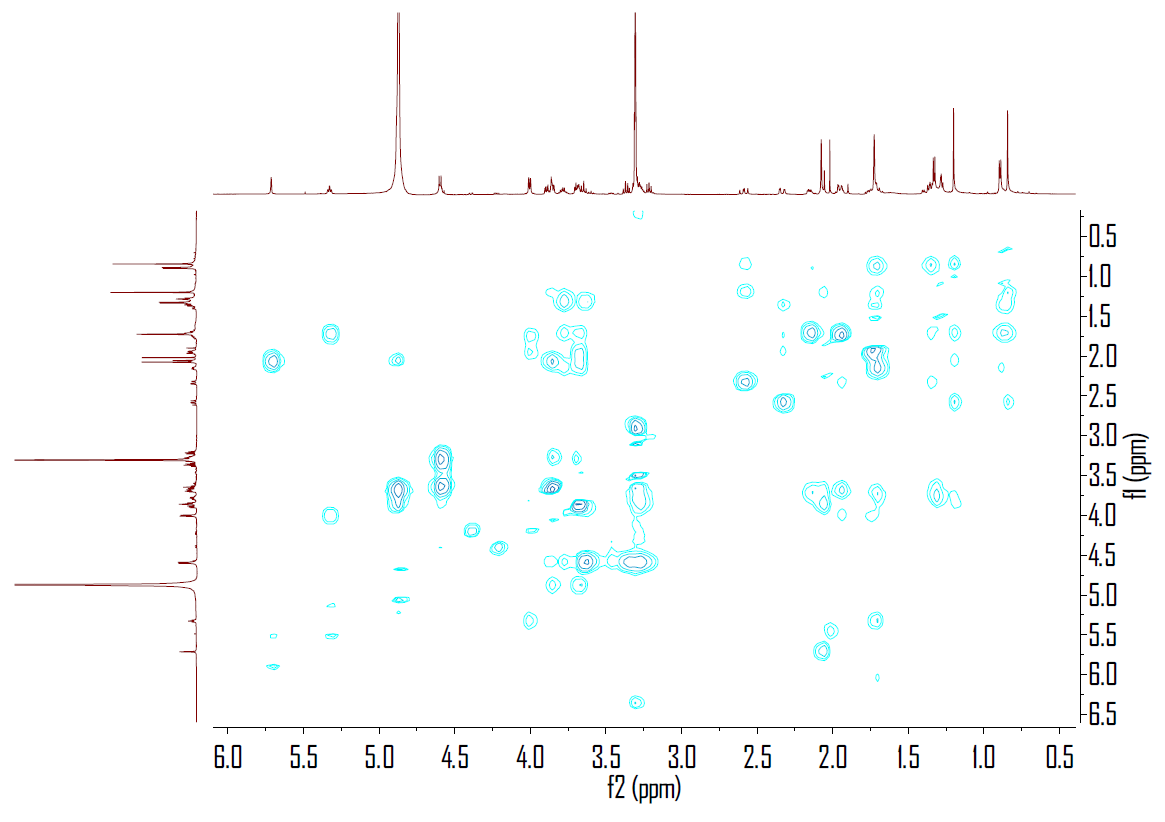


**Figure S24**. ROESY Spectrum (CD_3_OD) of compound **3**


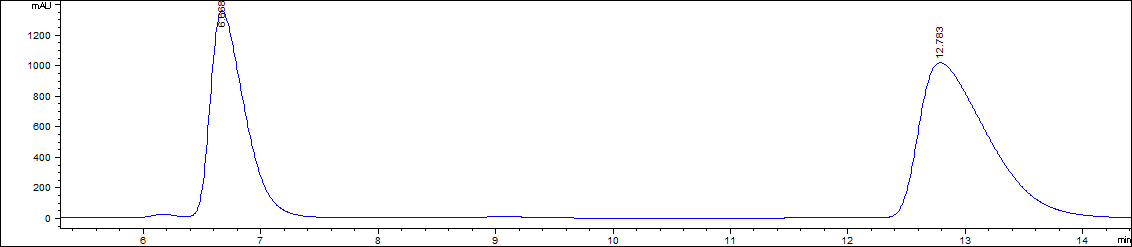


**Figure S25**. The retention time of Rha and Glc derivatives in HPLC (DAD, sig = 254nm)
